# Lead Drives Complex Dynamics of a Conjugative Plasmid in a Bacterial Community

**DOI:** 10.3389/fmicb.2021.655903

**Published:** 2021-05-28

**Authors:** Valentine Cyriaque, Jonas Stenløkke Madsen, Laurence Fievez, Baptiste Leroy, Lars H. Hansen, Fabrice Bureau, Søren J. Sørensen, Ruddy Wattiez

**Affiliations:** ^1^Laboratory of Proteomics and Microbiology, Research Institute for Biosciences, University of Mons, Mons, Belgium; ^2^Section of Microbiology, Department of Biology, University of Copenhagen, Copenhagen, Denmark; ^3^Cellular and Molecular Immunology Service, GIGA Research, University of Liège (ULG), Liège, Belgium; ^4^Section for Microbial Ecology and Biotechnology, Department of Plant and Environmental Sciences, University of Copenhagen, Frederiksberg, Denmark

**Keywords:** plasmid spread, metal, lead, conjugation, plasmid-mediated resistance, proteomics

## Abstract

Plasmids carrying metal resistance genes (MRGs) have been suggested to be key ecological players in the adaptation of metal-impacted microbial communities, making them promising drivers of bio-remediation processes. However, the impact of metals on plasmid-mediated spread of MRGs through selection, plasmid loss, and transfer is far from being fully understood. In the present study, we used two-member bacterial communities to test the impact of lead on the dispersal of the IncP plasmid pKJK5 from a *Pseudomonas putida* KT2440 plasmid donor and two distinct recipients, *Variovorax paradoxus* B4 or *Delftia acidovorans* SPH-1 after 4 and 10 days of mating. Two versions of the plasmid were used, carrying or not carrying the lead resistance *pbr*TRABCD operon, to assess the importance of fitness benefit and conjugative potential for the dispersal of the plasmid. The spread dynamics of metal resistance conveyed by the conjugative plasmid were dependent on the recipient and the lead concentration: For *V. paradoxus*, the *pbr* operon did not facilitate neither lead resistance nor variation in plasmid spread. The growth gain brought by the *pbr* operon to *D. acidovorans* SPH-1 and *P. putida* KT2440 at 1 mM Pb enhanced the spread of the plasmid. At 1.5 mM Pb after 4 days, the proteomics results revealed an oxidative stress response and an increased abundance of pKJK5-encoded conjugation and partitioning proteins, which most likely increased the transfer of the control plasmid to *D. acidovorans* SPH-1 and ensured plasmid maintenance. As a consequence, we observed an increased spread of pKJK5-*gfp*. Conversely, the *pbr* operon reduced the oxidative stress response and impeded the rise of conjugation- and partitioning-associated proteins, which slowed down the spread of the *pbr* carrying plasmid. Ultimately, when a fitness gain was recorded in the recipient strain, the spread of MRG-carrying plasmids was facilitated through positive selection at an intermediate metal concentration, while a high lead concentration induced oxidative stress with positive impacts on proteins encoding plasmid conjugation and partitioning.

## Introduction

Metals constitute a serious risk for ecosystems because of their biotoxicity and bioaccumulation. Previous studies have revealed that metals impact microbial communities by modifying their composition (Gillan et al., 2005; Guo et al., 2019), decreasing their diversity (Sun et al., 2013; Kwon et al., 2015; Guo et al., 2019), and/or reducing the activity rates of their members (Jurburg et al., 2017). However, after a long-term metal contamination, microbial communities proved to be resilient. In several cases, long-term metal exposure had no visible impacts on alpha diversity or activity (Gillan et al., 2015; Ni et al., 2016; Jacquiod et al., 2018; Cyriaque et al., 2020a). Horizontal gene transfer, especially when mediated by plasmids, was proposed as a mechanism involved in the resilience of microbial communities in metal-impacted ecosystems (Jacquiod et al., 2018; Cyriaque et al., 2020b). Mobile genetic elements thus seem to bridge clinical and environmental ecosystems, where metal resistance genes (MRGs) are associated (*via* cross- and co-resistance systems) with antibiotic resistance genes (ARGs), a major global threat to public health (Perry and Wright, 2013; Pal et al., 2015; Nishida and Oshima, 2019). Plasmids are considered key players in bacterial adaptation, as they mobilize genes contributing to genome innovation (Norman et al., 2009). Their persistence in a community is dependent on (i) their acquisition rate (conjugation and transformation), (ii) their fitness (i.e., ability to survive in a competitive environment) cost/benefit on their host, and (iii) loss rate (stability) (Bahl et al., 2009; Lopatkin et al., 2017). Furthermore, the fitness effect of the plasmid may also depend on specific genetic interactions between the plasmid and the rest of the host genome as demonstrated across *Pseudomonas* species in a mercury-selective environment (Kottara et al., 2018). Therefore, drawing a general scheme on the role of a metal as a selection factor for the maintenance and spread of plasmids encoding MRGs in a microbial community is not trivial. In *Cupriavidus metallidurans* CH34, metals increased the abundance of conjugative transfer proteins (Monchy et al., 2007), and cadmium was shown to increase plasmid dispersal in subsurface-derived sediment microcosms (Smets et al., 2003; Pu et al., 2021). Copper can either decrease conjugation frequency (Parra et al., 2019) or promote plasmid-mediated gene transfer (Zhang et al., 2019). Furthermore, metals were shown to decrease plasmid dispersal in a soil microbial community without impacting the diversity of transconjugants (Klümper et al., 2017). Metals either negatively or positively modulate plasmid uptake depending on the metal, metal concentration, and recipient, with a large variability between metals and metal concentrations (Klümper et al., 2017; Wang et al., 2020). Bio-remediation processes through plasmid-mediated MRG bio-augmentation rely on the spread of the plasmid in a metal-contaminated environment (Garbisu et al., 2017). Additional knowledge is needed to understand the impact of metals on selection, plasmid maintenance, and conjugation processes. The present work focuses on the effects of lead on the spread of a broad-host-range plasmid encoding a lead resistance system into a recipient population. Our initial hypothesis was that the plasmid-encoded lead resistance system would facilitate the spread of the plasmid in a metal-impacted environment.

**FIGURE 1:**
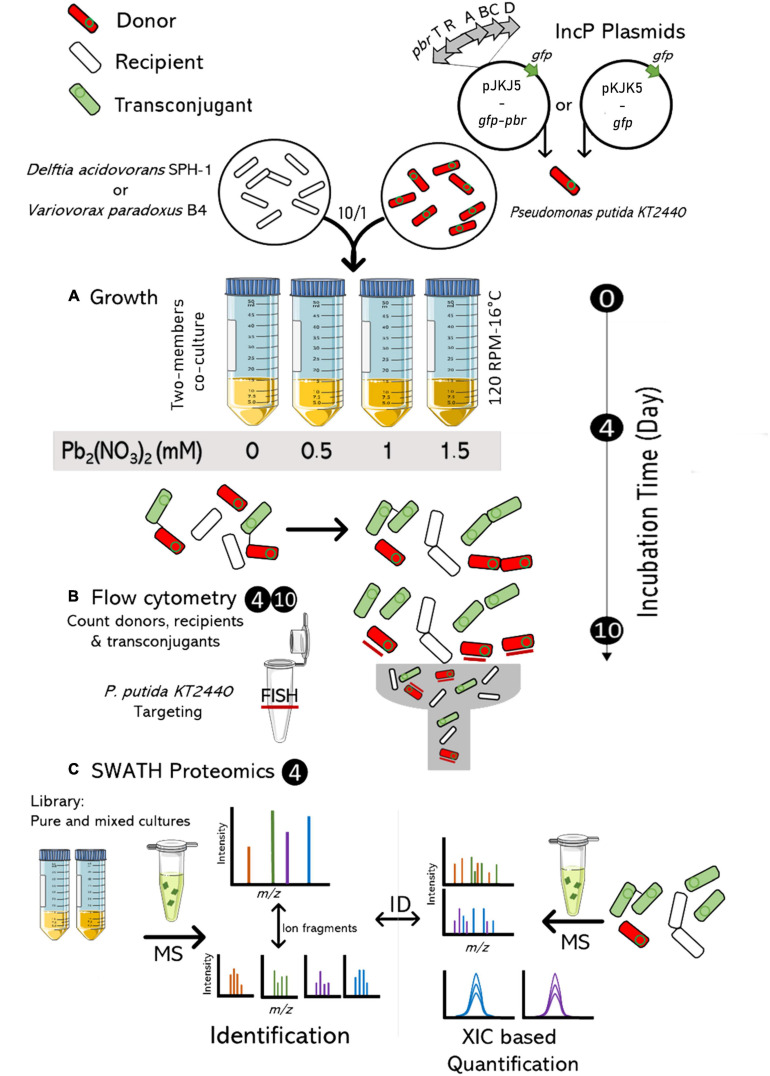
Experimental design combining flow cytometry and proteomics to assess lead impact on the functional profile of a mating coculture and the spread of the plasmid. Recipient and donor cells were grown (10/1 ratio) in LB3D (120 RMP, 16°C) over 10 days with an increasing concentration of Pb_2_(NO_3_)_2_ (0, 0.5, 1, 1.5 mM) **(A)**. After 4 and 10 days, flow cytometry was used to quantify recipient, donor (FISH labelled) and transconjugant cells **(B)**. After 4 days, they were also analyzed by SWATH-MS proteomics **(C)**. MS: Mass spectrometry; *m/z*: mass-to-charge ratio; XIC: extracted ion chromatogram.

Lead is a non-essential metal and a major metal pollutant in the environment with strong adverse effects. We investigated the impact of lead [Pb(II)] on the spread of a conjugative IncP-1ε plasmid in a recipient population carrying, or not, the lead resistance operon *pbrTRABCD* from *C. metallidurans* CH34. The spreads of pKJK5-*gfp* and pKJK5-*gfp*-*pbr* were investigated between a plasmid donor *Pseudomonas putida* KT2440 and a recipient strain. Several species identified in both non-contaminated and metal-contaminated (including lead) sediment (Gillan et al., 2015) were tested as recipient strains. *Variovorax paradoxus* B4 and *Delftia acidovorans* SPH-1 were chosen because a significant amount of transconjugants was obtained, because the pKJK5 plasmid could be easily introduced by electroporation for burden assays, and because these strains have a growth rate similar to the donor *P. putida* KT2440, limiting the impact of native growth rate on plasmid spread. The spread of the plasmid in the recipient pool was quantified, and the molecular adaptative response of plasmid donors and recipients was studied at increasing lead concentrations (Pb-0, Pb-0.5, Pb-1, and Pb-1.5 mM – Figure 1). To take the effect of the *pbr* operon on the fitness of the host into account, we monitored plasmid-free and plasmid-carrying cells after 4 and 10 days of co-culture. The long-term conjugation assay did not report a direct transfer from the donor cells to the recipients but instead the stability of the plasmid in the recipient community which is the sum of plasmid loss and gain as well as host fitness loss or gain. The involvement of these factors was deciphered by measuring the fitness effect of the plasmids on each strain and profiling the proteome of the mating co-cultures.

## Materials and Methods

### Strains, Plasmids, and Growth Conditions

For the construction of plasmid pKJK5-*gfpmut3-pbr*TRABCD-*kan*R-*tet*R, *C. metallidurans* CH34 and Electrocomp^TM^ GeneHogs^®^ *Escherichia coli* (Invitrogen) were cultivated in Luria–Bertani (LB) medium (see “Supplementary Method” for details on how the *pbr*TRABCD operon was inserted into pKJK5). The strain *P. putida* KT2440/Plpp*mCherry*-*kan*R harboring the plasmid pKJK5-*gfpmut*3-*kan*R-*tet*R (pKJK5-*gfp*) or pKJK5-*gfpmut*3-*pbr*TRABCD-*kan*R-*te*tR (pKJK5-*gfp-pbr*) was used as the plasmid donor. Precultures of those strains were grown in threefold diluted LB broth buffered with MOPS (2.1 g/L) (LB3D; peptone 3.3 g/L, NaCl 3.3 g/L, YE 1.7 g/L, MOPS 2.1 g/L) and supplemented with 50 μg/ml tetracycline (30°C, overnight). *V. paradoxus* B4 (DSMZ, Germany) and *D. acidovorans* SPH-1 (DSMZ, Germany) were used as plasmid recipients. An overview of these strains’ characteristics is displayed in Supplementary Table 1. Precultures of these strains were grown overnight in 457 medium (DSMZ) supplemented with 2 g/L mercaptosuccinate and LB3D (30°C). To assess the impact of the plasmid on the growth of its host, both plasmid donors and the clones *V. paradoxus* B4/pKJK5-*gfp*, *V. paradoxus* B4/pKJK5-*gfp-pbr*, *D. acidovorans* SPH-1/pKJK5-*gfp*, and *D. acidovorans* SPH-1/pKJK5-*gfp*-*pbr* were obtained by conjugation between those recipient cells and GeneHogs^®^ *E. coli*-bearing plasmids (“Supplementary Method”).

### Assessing Plasmid Fitness Effect

The fitness effect of plasmids pKJK5-*gfp* and pKJK5-*gfp-pbr* when carried by *P. putida* KT2440/PlppmCherry-*kan*R, *V. paradoxus* B4, or *D. acidovorans* SPH-1 was measured relatively to the corresponding plasmid-free strain as the ratio between the growth rate of plasmid-free and plasmid-carrying cells (μ0/μ1) (Lopatkin et al., 2017) considering that pKJK5 displays a low rate of plasmid loss (Bahl et al., 2004). Plasmid-carrying clones were obtained by conjugation as described in the “Supplementary Method” and selected on tetracycline. Second precultures in 100 ml LB3D (30°C) (supplemented with tetracycline 50 μg/ml for plasmid-carrying strains) were grown until an optical density (OD, 600 nm) between 0.4 and 0.6, measured with a Helios Zeta UV–vis spectrophotometer (Thermo Fisher Scientific). Then, the precultures were washed twice in LB3D (2 min, 7,000 × *g*), and cells were counted using a Bright-Line^TM^ Hemacytometer (Merck) following the manufacturer’s instructions. A stated number of cells (Supplementary Table 2) were sampled from precultures and diluted in a final volume of 15 ml LB3D supplemented with 0, 0.5, 1, or 1.5 mM Pb(NO_3_)_2_ in 50 ml falcon (*n* = 3) and incubated (16°C, 120 RPM) until the stationary phase. The OD (595 nm) was measured in 96-well plates multiplied with FLUO star OPTIMA (BMG LABTECH). Growth rate (*μ*) was quantified by log-transforming the growth curves and fitting the linear portion to obtain the slope of the regression (Supplementary Table 3). The significance of the fitness effect for each strain at different lead concentrations was assessed by comparing their growth rate with the plasmid-free corresponding strain.

### Plasmid Spread Assay in Liquid Community and Cytometry Analysis

The spreads of plasmids pKJK5-*gfp* and pKJK5-*gfp-pbr* carried by *P. putida* KT2440/Plpp*mCherry*-*kan*R as plasmid donor were assessed in co-culture with *V. paradoxus* B4 or *D. acidovorans* SPH-1 (Figure 1) with the equation *T* / (*DR*), where *D* is the count of donors (plasmid-carrying *P. putida* KT2440/Plpp*mCherry*-*kan*R), *R* is the count of recipients (plasmid-free *V. paradoxus* B4 or *D. acidovorans* SPH-1 cells), and *T* is the count of transconjugants (recipient cells that acquired the plasmid) (Jacquiod et al., 2017). In this long-term mating experiment, the recipient × donor normalization did not portray transfer frequency but instead a spread index of long-term plasmid transfer, plasmid loss, and a selection dynamics. Donor normalization was done as donor cells act as a plasmid source–sink (Hall et al., 2016) whose numbers, determined by *P. putida* KT440 growth, impact the plasmid invasion rate.

To start co-cultures, the strains were first grown separately in 100 ml LB3D (30°C; supplemented with 50 μg/ml tetracycline for donor strains) until an OD (600 nm) between 0.4 and 0.6 was measured using a Helios Zeta UV–vis spectrophotometer (Thermo Fisher Scientific). Then, bacteria were washed twice in LB3D (2 min, 7,000 × *g*), and cells were counted using a Bright-Line^TM^ Hemacytometer (Merck) following the manufacturer’s instructions. A stated number of cells (Supplementary Table 2) were sampled from the preculture and diluted in a final volume of 15 ml LB3D supplemented with 0, 0.5, 1, or 1.5 mM Pb(NO_3_)_2_ in 50-ml falcon (*n* = 3). Co-cultures were grown at 16°C and 120 RPM for 10 days and sampled (i) after 4 days for cell count and proteomics analyses (section “MRM Identification and Quantification of the PbrA Protein” and “Proteomic Analysis by SWATH MassSpectrometry”) and (ii) after 10 days for cell count (Figure 1). Donor, recipient, and transconjugant counts were assessed by flow cytometry for the detection of green fluorescent protein (GFP) fluorescence expressed by the *gfpmut3* gene carried by the pKJK5 plasmid carried by donor and transconjugant cells as described previously (Klümper et al., 2015; Cyriaque et al., 2020b). Plasmid donor cells were differentiated from transconjugants by flow cytometry (FC)-associated fluorescence *in situ* hybridization (FISH) targeting the 16S rRNA of *P. putida* as previously used (Gougoulias and Shaw, 2012). To that end, 1 ml of co-culture was centrifuged (6 min, 6,000 × *g*) and fixed by resuspending it in 1 ml PFA (4%), pH 7, for 15 min. The fixed cells were washed twice in PBS (6 min, 6,000 × *g*), resuspended in 247 μl of prewarmed (48°C) hybridization buffer supplemented with 3 μl of the probe PSE1284 (Yamaguchi et al., 2006) associated with an Alexa Fluor 647 fluorochrome (Eurogentec, Liège, Belgium) (250 ng/μl), and incubated for 4 h. The samples were centrifuged (5 min, 16,000 × *g*) and resuspended in 500 μl of hybridization buffer for 20 min. They were centrifuged (5 min, 16,000 × *g*) again and resuspended in 500 μl of wash buffer. Finally, the samples were centrifuged, resuspended in 1 ml PBS, and stored at 4°C until cytometry analyses. The hybridization [urea (1 M), NaCl (0.9 M), Tris HCl (pH 7.4, 20 μM)] and wash [urea (4 M), NaCl (0.9 M), Tris HCl (pH 7.4, 20 μM)] buffers contained urea as a denaturation agent to avoid GFP denaturation as described previously (Lawson et al., 2012; Kommerein et al., 2017). Cytometry analyses were carried out using a BD Science FACS Fortessa in a 96-well microplate with the following parameters: forward scatter, 390 V; side scatter, 176 V; detectors for green fluorescence associated with GFPmut3 (bandpass filter 530/30 nm, 501 V) and for red fluorescence associated with Alexa Fluor 647 (bandpass filter 670/14 nm, 550 V). FlowJo V10 was used to analyze the results to count donors, empty donors, recipients, and transconjugants (Supplementary Table 4). To confirm the accuracy of FC-FISH and GFP integrity, pure cultures of each donor and recipient were tested in upstream mating experiments (Supplementary Figure 1).

### Proteomic Analysis by SWATH Mass Spectrometry

A quantitative proteomic approach was used to assess the metal’s impact on plasmid transfer machinery and bacterial proteomes after 4 days of mating, concurrently with plasmid dispersion measurement. For that, 600 μl of 0 and 0.5 mM lead-cultured samples and 1,200 μl of 1 and 1.5 mM lead-cultured samples were harvested, centrifuged, and washed twice (6,000 × *g*, 6 min, 4°C) with PBS. The proteins were extracted, reduced, alkylated, precipitated, and trypsinized from the pellet using the PreOmics Kit (PreOmics GmbH, Germany), following the manufacturer’s instructions. The obtained peptides were quantified using the Pierce^TM^ Quantitative Colorimetric Peptide Assay (Thermo Fisher Scientific). For post-acquisition retention time calibration, a PepCalMix solution (Protein Extract Digest) (AB SCIEX, Framingham, MA, United States) was added to 4 μg of peptides (50 fmol on column) following the manufacturer’s instructions. The peptides (2 μg on column) were analyzed on a UHPLC-HRMS/MS instrument (AB SCIEX LC420 and TripleTOF^TM^ 6600) using SWATH data-independent acquisition as described in the “Supplementary Method.” SWATH wiff files were processed using AB SCIEX PeakView 2.2 software and SWATH^TM^ Acquisition MicroApp. Up to six peptides with at least 95% confidence were selected, with six transitions per peptide. The XIC extraction window was set to 15 min, and the XIC width was set to 70 ppm. The peptide area corresponds to the sum of the fragment ion area, and the protein area corresponds to the sum of the peptide area. The protein areas were extracted and exported in AB SCIEX MarkerView^TM^ 1.2 software for normalization and statistical analysis. The protein extraction characteristics, the number of proteins identified in both libraries at 1% FDR, and the proteome coverage of the SWATH proteomic analyses are displayed in Supplementary Tables 5, 6. To be able to compare the different samples, protein relative abundances were obtained by dividing each protein abundance by the cumulated protein area of the corresponding sample. The obtained dataset underwent a second normalization: (i) chromosomally encoded proteins were normalized by the total protein abundance associated with the specific strain. We used the protein biomass (and not cell count) to normalize protein counts because the expression level of proteins per cell depends on the strain (Cortes et al., 2019). Therefore, using cell count as a normative factor unbalanced the protein counts (Supplementary Figures 2–5); and (ii) for plasmid-encoded proteins, it was impossible to discriminate what bacterium expressed the proteins. In this case, protein counts were instead normalized by the proportion of plasmid-carrying cells in the two-member community obtained by flow cytometry. The results were deposited on the Peptide Atlas public repository^1^ under accession number PASS01468^2^. Significant differences between norm2-protein abundances (log-2-transformed) were determined using a two-tailed Student’s *t*-test across the different lead concentrations for each filter mating association and for the comparison of the same lead condition impacting the mating pair with or without *pbr*TRABCD in the exchanged plasmid. Proteins displaying a minimum *p*-value below 0.05 were taken into consideration, and strain-specific proteins with a *p*-value below 0.01 were plotted in heat maps using hierarchical cluster dendrograms (Euclidean distance and average clustering) in RGui software [vegan (Oksanen et al., 2019), rioja (Juggins, 2019), and gplots (Warnes et al., 2016) R-packages]. The strain-specific proteins are displayed in the “Heat Map Supplementary Material.”

### MRM Identification and Quantification of the PbrA Protein

Quantification of PbrA was performed using multiple reaction monitoring (MRM)-based relative quantification. The spectral signature of PbrA was obtained through the analysis of *C. metallidurans* CH34 samples using a regular liquid chromatography–tandem mass spectrometry (LC–MS/MS) procedure (Leroy et al., 2015). This sample was selected because *C. metallidurans* is known to express, in the presence of a high lead load, a high level of PbrA. Bacterial culture was obtained as described previously (30), proteins were extracted and digested using the PreOmics Kit (PreOmics GmbH, Planegg/Martinsried, Germany), and peptides were quantified using a Pierce^TM^ Quantitative Colorimetric Peptide Assay (Thermo Fisher Scientific) following the manufacturer’s instructions. A total of 13 peptides were detected in the LC–MS/MS analysis of *C. metallidurans* and evaluated for quantification in MRM mode on a Q-Trap 6500+ coupled to LC420 chromatography (SCIEX) operated in microflow mode. After transition optimization and interference removal, four peptides with four to five transitions each were selected for quantification (Supplementary Table 5). Quantification was assessed on (i) the pKJK5-*gfp*-carrying sample with 1 mM lead as a control and (ii) all pKJK5-*gfp-pbr*-carrying samples. For that, 3 μg of trypsin-digested proteins was separated on a C18 YMC-Triart 0.3 × 150-mm column operated at a flow rate of 5 μl/min with an ACN gradient from 2 to 35% in 17 min. Skyline was used for visual inspection of the data and area under the curve integration. Peak picking for each peptide was manually refined using the transition intensity ratio and retention time as leading parameters. These parameters were obtained from a *C. metallidurans* sample that contained a higher level of PbrA. The intensity of all four to five transitions was summed up for each peptide. Protein abundance was obtained as the average of the Ln-transformed area under the curve of each of the four peptides.

## Results

### Fitness Effect of Plasmids on Their Host

**FIGURE 2:**
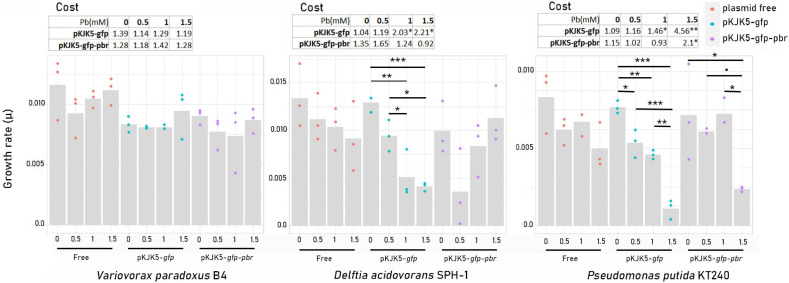
Growth rates (*μ*) and plasmid fitness effect (cost, *μ*_0_ / *μ*_1_) ± SEM, where *μ*_0_ = growth rate of the plasmid-free cells and *μ*_1_ = growth rate of plasmid-carrying cells in pure cultures. The stars show significant differences among growth rates and the significance of the fitness effect (comparison between growth rates of the plasmid-carrying cells and plasmid-free cells at the same lead concentration). *p-value < 0.05; **p-value < 0.01; ***p-value < 0.001 (*N* = 3) (*n* = 3; Tukey test; *p* ≤ 0.05).

The fitness effect of the pKJK5-*gfp* and pKJK5-*gfp*-*pbr* plasmids impeding the growth of plasmid donors and recipients was calculated as the ratio of the growth rate of plasmid-free and plasmid-carrying cells. Without lead exposure, none of the plasmids had any significant fitness effect on *P. putida* KT2440, *D. acidovorans*, or *V. paradoxus* B4 (Figure 2).

In the presence of lead, neither pKJK5-*gfp* nor pKJK5-*gfp-pbr* had a cost for *V. paradoxus* B4. We, therefore, expected no lead-mediated positive selection of the plasmids into *V. paradoxus* B4.

However, the growth rate of *D. acidovorans* SPH-1/pKJK5-*gfp* was significantly decreased at high lead concentrations (Pb-1 and Pb-1.5). This fitness effect was compensated by the *pbrTRABCD* operon, dedicated to lead resistance. Despite being non-significant, the growth rate of *D. acidovorans* SPH-1 carrying pKJK5-*gfp-pbr* showed a decreasing trend in Pb-0.5. At low lead concentrations, the cost imposed by the *pbr* operon would then be larger than the benefit it procured to the host. Lead had a negative impact on the growth rate of plasmid carrier *P. putida* KT2440/pKJK5-*gfp*. In Pb-0.5 and Pb-1, the *pbr* operon compensated for the negative fitness effect of the plasmid. In Pb-1.5, decreased growth was significant in both plasmid carriers, with an attenuated effect on pKJK5*-gfp-pbr*-carrying cells (Figure 2).

### Two-Member Community Dynamics Assessed by Flow Cytometry

**FIGURE 3:**
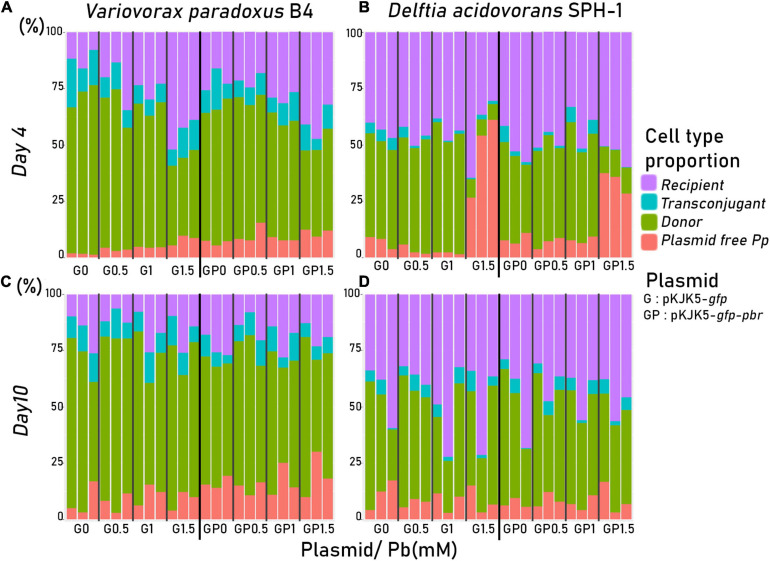
Cell proportions measured among 30,000 cells after 4 days **(A,B)** or 10 days **(C,D)** of co-culture using a BD Science FACS Fortessa flow cytometer. Plasmid-carrying cells were detected by green fluorescence associated with GFPmut3. *Pseudomonas putida* KT2440 was identified by flow-FISH using an Alexa Fluor 647 fluorescent specific probe. The associated spread index is displayed in Figure 4.

**FIGURE 4:**
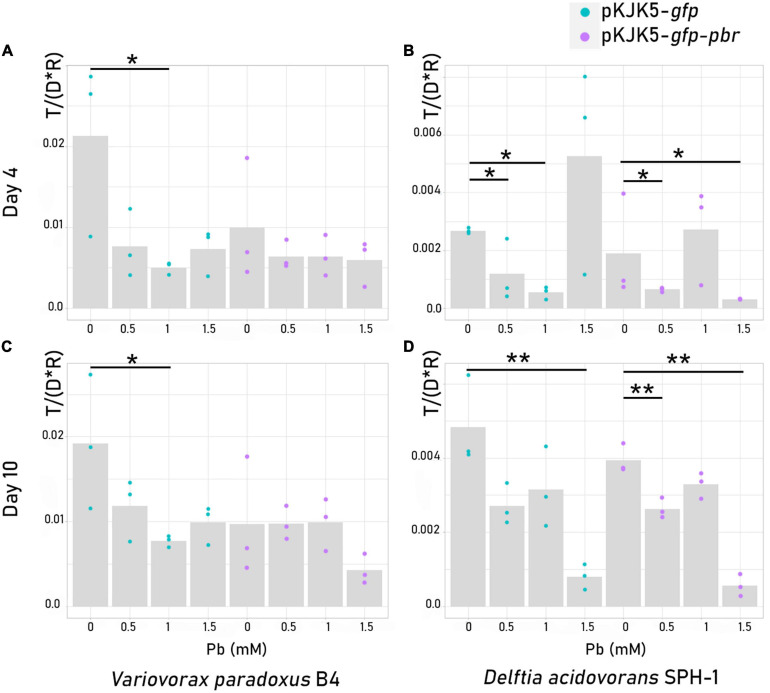
Plasmid spread index [*T* / (*D* * *R*)] ± SEM in recipient cells, *Variovorax paradoxus* B4 **(A,C)** or *Delftia acidovorans* SPH-1 **(B,D)**, after 4 days **(A,B)** or 10 days **(C,D)** of mating. The *p*-values were calculated from Kruskal–Wallis tests (4-day mating) or log-2-transformed abundances using *t*-tests (10-day mating) depending on homoscedasticity. *p-value < 0.05; **p-value < 0.01; ***p-value < 0.001 (*N* = 3). The dotted lines are for pKJK5-*gfp* comparisons, and the continuous lines are for pKJK5-*gfp-pbr* comparisons.

The proportion of donors (*P. putida* KT2440), recipients, and transconjugants in a two-member mating assay was measured using flow cytometry (Figures 1, 3 and Supplementary Table 4). We used either *D. acidovorans* SPH-1 or *V. paradoxus* B4 as recipient strains. Plasmid spread was detected in both recipients, with a higher frequency in *V. paradoxus* B4 (Figures 3, 4). For this strain, lead only had a significant negative impact on the spread of pKJK5-*gfp* in the recipient community in Pb-1 after both 4 and 10 days.

When *D. acidovorans* SPH-1 was the recipient strain, after 4 days, Pb had a negative impact on pKJK5-*gfp* dispersion until Pb-1. Interestingly, the spread of pKJK5-*gfp* in Pb-1.5 was not different from that in the control (Pb-0) after 4 days of mating, but the transconjugants relapsed after 10 days. After 4 days, in Pb-1, the spread of pKJK5-*gfp-pbr* was not impacted as opposed to pKJK5-*gfp*.

When *V. paradoxus* B4 was used as the recipient, plasmid loss in the donor cell fraction (plasmid-free *P.p*.; Figure 3 and Supplementary Table 4) progressively increased with lead concentration and time. When *D. acidovorans* SPH-1 was used as the recipient, plasmid loss in the donor *P. putida* KT2440 was high in Pb-1.5 where the fitness effect of both plasmids was the highest (the percentage of cured cells was calculated as the ratio between “empty *P.p.*” and total “*P.p.*”: 84.3 ± 4.2% of cured cells from pKJK5-*gfp* and 74.1 ± 1.4% from pKJK5-*gfp-pbr*) after 4 days of mating, but plasmid-free *P. putida* KT2440 largely decreased after 10 days (18.5 ± 12.6% of cured cells from pKJK5-*gfp* and 11.4 ± 3.1% from pKJK5-*gfp-pbr*) (Figure 3 and Supplementary Table 4).

### Meta-Proteomic Profiling of Two-Member Communities

To obtain a better understanding of the functional response to lead in the co-cultures, a quantitative meta-proteomic analysis was performed using a SWATH approach. A spectral library built with a data-dependent acquisition (DDA) workflow was generated with monocultures and co-cultures of donors and recipients (Supplementary Table 2) at 0 and 1 mM Pb(NO_3_)_2_. The label-free DDA analyses of pure cultures revealed that lead was associated with metal resistance proteins (Supplementary Table 6). In *P. putida* KT2440, we observed the induction of phosphate metabolism-associated proteins (e.g., phosphatase) and pyoverdine-associated proteins. The ATPase PbrA was exclusively observed in *P. putida* KT2440/pKJK5-*gfp-pbr* in the presence of lead. In *V. paradoxus* B4, a high level of TonB siderophore-related proteins and a putative ABC transporter iron-binding protein and the iron-sulfur assembly scaffold protein IscU were upregulated. In *D. acidovorans* SPH-1, a large diversity of metal resistance-associated proteins was observed, including (i) phosphate metabolism-associated proteins, (ii) TonB siderophore-related proteins, (iii) efflux pumps, (iv) glutathione S-transferase, (v) the iron–sulfur assembly protein IscA, (vi) thioesterase, (vii) iron permease, and (viii) bacterioferritin (Supplementary Table 6). Using SWATH-MS results, the relative biomass of the donor and recipient bacteria in co-cultures was evaluated as the sum of the area of all proteins attributed to the specific strain (Supplementary Table 4). The coverage of the proteome of each bacterial member is displayed in Supplementary Table 7.

#### Differential Impacts of Lead on Metabolic Response and Resistance Mechanisms

When the plasmid donor *P. putida* KT2440 was grown in the presence of *V. paradoxus* B4, a total of 632 proteins assigned to *P. putida* KT2440 were significantly impacted by lead (*p-*value < 0.05; 233, *p-*value < 0.01). A total of 734 proteins assigned to *V. paradoxus* B4 were significantly impacted by lead (*p-*value < 0.05; 296, *p-*value < 0.01). When the *P. putida* KT2440 plasmid donor was grown in the presence of *D. acidovorans* SPH-1, a total of 739 proteins were significantly impacted by lead (*p-*value < 0.05; 352, *p-*value < 0.01). The abundance of proteins identified as belonging to *D. acidovorans* SPH-1 that were significantly impacted by lead (585, *p-*value < 0.05; 254, *p-*value < 0.01) was increased the most at a lead concentration of 1.5 mM.

**FIGURE 5:**
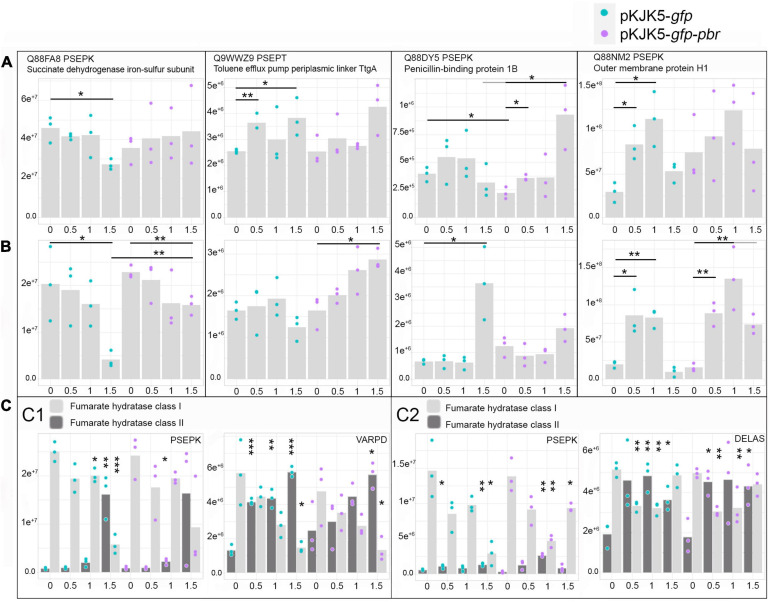
Abundances (± SEM) of proteins of interest in the two-member community without the *pbr*TRABCD operon (blue) or including the *pbr*TRABCD operon (purple) depending on Pb(II) concentration (mM). Different panels represent the abundance of proteins identified as belonging to *Pseudomonas putida* KT2440 when cultivated with *Variovorax paradoxus* B4 **(A)** or with *Delftia acidovorans* SPH-1 **(B)**; the abundance of fumarate hydratase enzymes in *P. putida* KT2440 (PSEPK) and *V. paradoxus* B4 (VARPD) when cultivated together (C1) and in *P. putida* KT2440 (PSEPK) and *D. acidovorans* SPH-1 (DELAS) when cultivated together (C2). The *p*-values were calculated from log-2-transformed abundances using a *t*-test (*n* = 3). *p-value < 0.05; **p-value < 0.01; ***p-value < 0.001. In **(C)**, the *p*-values were calculated between the different lead concentrations (Pb0.5, Pb1, or Pb1.5) and the control (Pb0) of the same co-culture.

The detailed metabolic modulation in the SWATH proteomes of the three strains in both mating pair conditions with increasing lead concentrations is displayed in the “Heat Map Supplementary Material.” In Pb-1.5, in co-cultures with *D. acidovorans* SPH-1, *P. putida* KT2440’s DNA repair proteins [transcription repair coupling factor (mfd), MutS, SbcC, L COG; “Heat Map Supplementary Material”] and stress proteins (CysQ; P COG, “Heat Map Supplementary Material”) were overabundant. Among lead-impacted proteins, enzymes requiring divalent cation cofactors [Fe(II), Mg(II), Zn(II), Co(II), and Mn(II)] or the [4Fe–4S] cluster were highly represented in Pb-1.5. Although lead cannot directly inactivate the [4Fe–4S]-dependent class I enzyme (Xu and Imlay, 2012), the oxidative stress that Pb most likely induced (Wang et al., 2012; Jarosławiecka and Piotrowska-Seget, 2014) decreased the abundance of these enzymes as previously shown with Al–Ga toxicity (Chenier et al., 2008). Among these enzymes, [4Fe–4S]-dependent fumarate hydratase class I was replaced by iron-independent fumarate hydratase class II (Figure 5C). When grown with *D. acidovorans* SPH-1, the class I enzyme of *P. putida* KT2440 was much less decreased in abundance at Pb1.5 when grown with the *pbr* operon. Similarly, the decreased abundance of the succinate dehydrogenase iron–sulfur subunit of *P. putida* KT2440 was amplified in the presence of *D. acidovorans* SPH-1 (Figures 5A,B) and reduced with the benefit of the *pbrTRABCD* operon. In *V. paradoxus* B4, an increased amount of pilus-related proteins (“Heat Map Supplementary Material”) may explain the large mobility of pKJK5 plasmids into this recipient strain compared with *D. acidovorans* SPH-1 (Figures 3, 4).

**FIGURE 6:**
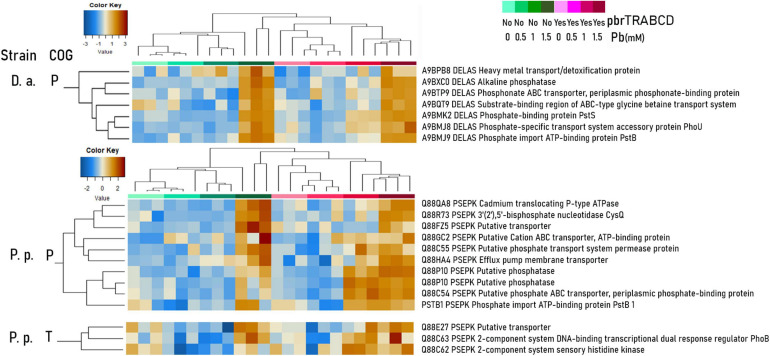
Centered-scaled log-2-transformed abundances of *Pseudomonas putida* KT2440 (P.p.) and *Delftia acidovorans* SPH-1 (D.a.) proteins (two or more identified peptides) of interest belonging to P (inorganic ion transport and metabolism) and T (signal transduction mechanisms) COGs are displayed in heat maps (Euclidean distance, average clustering; entire heat maps: see “Heat Map Supplementary Material”).

At high lead concentrations, all strains upregulated metal resistance-associated proteins involving phosphatases (production of phosphate salt for the precipitation of metal cations; Jarosławiecka and Piotrowska-Seget, 2014), metabolism of glutathione (binding metal cations; Taghavi et al., 2009), and efflux transporters (RND efflux systems and heavy-metal transporters; P COG) (“Heat Map Supplementary Material”). In the *V. paradoxus* B4 proteome, additional siderophore-related proteins were also overabundant, such as TRAP dicarboxylate transporters, TonB receptors (P COG), a putative non-ribosomal peptide synthase (NRPS), and polyketide synthase (Q COG), for the biosynthesis of variochelin lipopeptide siderophores (Kurth et al., 2016). In *D. acidovorans* SPH-1, a large number of metal resistance-induced proteins were overabundant, including TonB siderophores, phosphatases, and efflux pumps, explaining its high fitness (P COG) (“Heat Map Supplementary Material”). *P. putida* KT2440 also upregulated pyoverdine-associated proteins (V COG) (Taghavi et al., 2009; Naik and Dubey, 2011; Jarosławiecka and Piotrowska-Seget, 2014). When *P. putida* KT2440 was co-cultured with *D. acidovorans* SPH-1 in Pb-1.5, (i) penicillin-binding protein 1B was overly increased, especially when carrying pKJK5-*gfp* (Figure 5B), and (ii) toluene efflux periplasmic linker protein TtgA and outer membrane protein H1 were increased exclusively when carrying pKJK5-*gfp-pbr* (Figure 5B). The outer membrane protein H1 replaces divalent cations at binding sites on lipopolysaccharide and would then prevent the uptake of Pb(II) ions (Bell and Hancock, 1989). In Pb-1, phosphatase, phosphate-binding and transport-associated proteins as well as putative cation transporter (P COG) and PhoB and PhoR proteins (T COG) were more abundant when the *pbrTRABCD* operon was present (Figure 6). Displayed resistance systems were then dependent on the strain, its mating partner, and the presence of the *pbr* operon.

#### Lead Impacts on pKJK5 Backbone Genes

**FIGURE 7:**
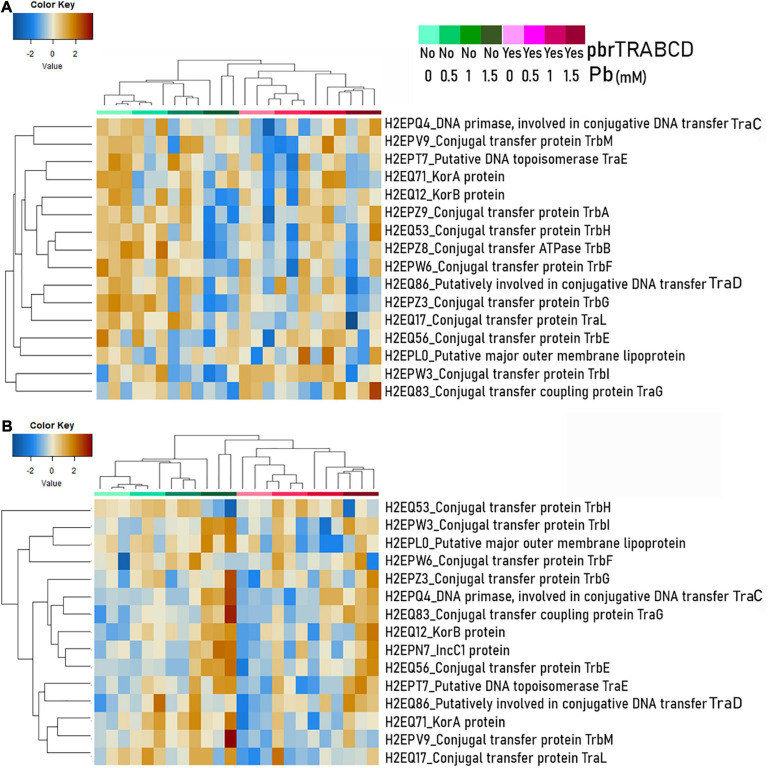
Abundance of conjugation-associated proteins (two or more identified peptides) encoded by the pKJK5 plasmid [normalization on the summed area of all peptides for each sample and by cell proportion of plasmid carrier (donor + transconjugant) (see Supplementary Table 8)] displayed on a heat map (Euclidean distance and average clustering) as extracted from two-member communities using either *Variovorax paradoxus* B4 **(A)** or *Delftia acidovorans* SPH-1 **(B)** as the plasmid recipient.

To decipher lead effects on the conjugation machinery, we also inspected conjugation-associated proteins encoded by the pKJK5 plasmids. We normalized the abundance of these proteins by the proportion of plasmid-carrying cells assessed by flow cytometry. When *V. paradoxus* B4 was the mating partner, 16 proteins encoded by the pKJK5 plasmid were significantly impacted. Among them, TraD, TrbA, TrbB, TrbF, TrbG, and TrbH were decreased at 1.5 mM (Supplementary Table 8 and Figure 7). When *D. acidovorans* SPH-1 was the mating partner, in Pb-1, TraC and TraG of the pKJK5-*gfp-pbr* plasmid displayed a positive fold-change. In Pb-1.5, the proteins KorB, IncC1, TraC, TraE, TraG, TrbE, and TrbI were increased, especially in pKJK5-*gfp* (Figure 7 and Supplementary Table 8).

#### Plasmid-Encoded PbrA Expression

Pbr proteins were not detected in the SWATH analysis, revealing their low abundance level. Therefore, PbrA relative abundance was measured for the samples carrying the pKJK5-*gfp*-*pbr* plasmid using a targeted MRM-based relative quantification approach (Supplementary Figure 6). This sensitive method revealed the presence of PbrA in the samples. No significant differences in the *V. paradoxus* B4 mating pair and an increased relative abundance in the *D. acidovorans* SPH-1 mating pair (at Pb 1 and 1.5 mM) were observed.

## Discussion

Previous works revealed that the presence of metals either favor (Smets et al., 2003; Monchy et al., 2007) or inhibit the spread of plasmids (Klümper et al., 2017; Parra et al., 2019) with different impacts on the bacterial members of a soil community (Klümper et al., 2017). It was suggested that the cost and advantage trade-offs imposed by plasmids make their dispersion a dynamic process (Pinilla-Redondo et al., 2018; Cyriaque et al., 2020b). The pKJK5 plasmid has been shown to ensure its stability in a bacterial population using a high conjugation efficiency (Bahl et al., 2007) and an analogous partitioning system to the RK2 plasmid (Rosche et al., 2000). Lead may either modulate the maintenance machinery or topple the cost/benefit ratio.

### Lead, Mating Partner, and MRGs Modulated the Fitness of the Hosts

*Pseudomonas putida* KT2440, *V. paradoxus* B4, and *D. acidovorans* SPH-1 separately displayed similar lead minimal inhibitory concentrations (MICs, 2 mM in LB 3D). Fitness effect was calculated as the ratio between the separate growth rate of plasmid-free and plasmid-carrying cells. Although the broad-host-range pKJK5 plasmid was shown to be stable in *Escherichia coli* (Bahl et al., 2004), a plasmid loss in a part of the population cannot be dismissed, especially since plasmid-free *P. putida* arose in the mating experiment, mostly after 4 days, at high lead concentration. If so, the fitness effect of the plasmid might have been underestimated. Nevertheless, some observations can be addressed: First, lead had no significant effect on the growth of any of the plasmid-free strains. Moreover, while lead had a significant impact on plasmid-bearing *D. acidovorans* SPH-1 and *P. putida* KT2440, no significant effect was recorded on the growth rate of plasmid-bearing *V. paradoxus* B4. The presence of the *pbr* operon had no effect on the growth of *V. paradoxus* B4 when exposed to lead, suggesting that the *pbr* operon is not beneficial for *V. paradoxus* B4. This might be due to the high upregulation of siderophore-related proteins (e.g., NRPS, polyketide synthase and outer-membrane TonB receptor). As a consequence, the *pbr* operon would then be obsolete in the resistance against lead. PbrA is an efflux pump transporting zinc, cadmium, and lead ions from the cytoplasm to the periplasm, where phosphates released in the periplasm by the undecaprenyl pyrophosphate phosphatase PbrB form metallophosphates with lead cations (Hynninen et al., 2009). This system would then compete with lead-binding siderophores. Consequently, plasmid spread in the *V. paradoxus* B4 population depends on conjugation and stability systems regardless of the MRGs it carries. In *D. acidovorans* SPH1 and *P. putida* KT2440, the *pbr* defense system decreased the fitness effect of the plasmid, impacting the spread of the plasmid in *D. acidovorans* SPH-1. After 4 days of mating, at Pb1, the spread of pKJK5-*gfp-pbr* was higher than pKJK5-*gfp*. Similar selection dynamics where plasmids provide a fitness benefit have been recorded many times in the presence of antibiotics (Lopatkin et al., 2016). Such selection processes and the colocation of ARGs and MRGs on plasmids may amplify the risk of spread of ARGs in metal-contaminated environments. After 10 days, the plasmid continued to spread and the pKJK5-*gfp* plasmid caught up with the *pbr* variant.

It should be noted that, in these co-cultures, the *P. putida* KT2440 proteome reveals that the presence of the *pbr* operon was associated with an overabundance of alternative systems. For instance, we see an overabundance of the outer membrane protein H1, the toluene efflux pump TtgA, phosphatases, and proteins associated with phosphate import, especially in Pb-1, where the dispersion of pKJK5-*gfp-pbr* was maintained, in contrast to pKJK5-*gfp*. Phosphatases and phosphate import-related proteins may be required to ensure the turnover of undecaprenyl pyrophosphate in the membrane associated with the *pbr* resistance system. Beneficial *pbr* genes then most likely sustained plasmid dispersion in Pb-1 after 4 days of mating by increasing the fitness of their host.

### Metal-Mediated Oxidative Stress at High Lead Concentrations Seemed to Have Consequences for the Stability of the Plasmids

At Pb 1.5, an increased number of plasmid-free *P. putida* KT2440 was recorded in co-cultures with *D. acidovorans* SPH-1, concomitantly to an increased cost of both plasmids for *P. putida* KT2440. This large fitness decrease most probably combined the negative effects on the growth rate imposed by the plasmid and the high lead concentration, while the *pbr* system was not efficient enough to completely alleviate the negative fitness effect of the plasmid. Surprisingly, when co-cultured with *V. paradoxus* B4, plasmid-free *P. putida* KT2440 cells did not increase drastically. As mentioned above, an up-regulation of siderophore-associated proteins of *V. paradoxus* B4 was observed. Siderophores are public goods – secreted molecules that benefit neighboring individuals – in this case by decreasing the bio-availability of the metal (Hesse et al., 2018). Siderophores might then have efficiently decreased the cumulated fitness effects of the plasmid and lead for both *V. paradoxus* B4 and *P. putida* KT2440.

The *pbr* operon alleviated the cost of the pKJK5 plasmid on *P. putida* KT2440 and the loss of pKJK5 in *P. putida* KT2440 when associated with *D. acidovorans* SPH-1. However, the number of plasmid-free cells was still very high. Interestingly, despite a reduction in potential plasmid donors, transconjugants were still detected among *D. acidovorans* SPH-1. The large spread of the pKJK5-*gfp* plasmid in the *D. acidovorans* SPH-1 recipient population at 1.5 mM lead after 4 days of co-culture concurred with the upregulation of partitioning- and conjugation-involved proteins (TraG, TraC, TrbI, TrbE, IncC1, and KorB), which most likely indicate an increased transfer and a decreased loss of the plasmid. IncC1 and KorB are part of the partitioning system (Rosche et al., 2000), T4CP and TraG are conjugative coupling proteins (T4CP, TraG) and the DNA primase TraC allows plasmid replication (Lanka and Barth, 1981; Bates et al., 1998; Schröder et al., 2002), ensuring the fertility of conjugation (Bates et al., 1998). The high increase in abundance of pKJK5-*gfp* conjugation proteins at Pb-1.5 concurs with signs of oxidative stress demonstrated by the negative impacts of the metal on iron–sulfur cluster-dependent proteins and the overabundant stress proteins (CysQ, MutS, SbcC, mdf) as previously shown (Zhang et al., 2019; Wang et al., 2020; Pu et al., 2021), whereas *pbr* genes most likely decreased this oxidative stress, subsequently reducing the promotion of partitioning- and conjugation-associated genes. In the long-term co-cultures (10 days), plasmid settlement was most likely determined by its fitness effect as plasmid occurrence in the recipient community decreased. Considering the potential effects of oxidative stress, the low fluctuation in the spread index of both plasmids into *V. paradoxus* might have been due to an inexistant stress, thanks to the previously mentioned involvement of siderophore-related proteins. As *pbr* brings no advantage to *V. paradoxus* B4, no fitness gain nor oxidative stress reduction would impact the spread of the plasmid.

These results unraveled factors impacting the plasmid-mediated spread of a MRG at high lead concentration in a two-member community *in vitro*, showing that associated metal differently impacted the spread of the pKJK5 plasmids. The metal-mediated oxidative stress modulated the spread of the plasmid by promoting the expression of conjugation and partitioning proteins. MRGs, if bringing any advantage to the host, would reduce the oxidative stress and (i) increase the fitness benefit of the plasmid but (ii) impede stress-activated conjugation/partitioning protein promotion, yet this assertion should be investigated on a shorter term to measure the actual effect of oxidative stress on plasmid transfer and its subsequent persistence in a strain population. Moreover, it was previously shown that host plasmid co-adaptation leads to a decreased plasmid cost, which might have influenced plasmid stability in the recipient pool over the 10 days of co-culture.

## Conclusion

In the present study, when a fitness gain was recorded in the recipient strain, the spread of MRG-carrying plasmids was facilitated through positive selection at an intermediate metal concentration. At high metal concentrations, while metal-mediated oxidative stress increased the abundance of proteins involved in conjugation/partitioning and facilitated the spread of the plasmid, the MRG curbed this oxidative stress and subsequently slowed down plasmid spread. Future studies will test a larger range of strains with different lead resistance potentials to increase the advantage brought by the plasmid-carried MRG, and the fine regulation of the conjugation machinery must be deciphered. Nonetheless, in light of these results, metals most likely influence the journey of plasmids in a diversified recipient community. These data thus represent valuable insights when considering plasmids for metal pollution bioremediation.

## Data Availability Statement

The datasets presented in this study can be found in online repositories. The names of the repository/repositories and accession number(s) can be found below: http://www.peptideatlas.org/, PASS01468.

## Author Contributions

VC and RW conceived the study. VC and LF performed the experiments and data analysis. JM and BL helped with experiments and assisted with computational analysis. LH, SS, FB, and RW contributed to reagents, materials, and analysis tools. VC, LF, JM, SS, and RW wrote the manuscript. All authors contributed to the article and approved the submitted version.

## Conflict of Interest

The authors declare that the research was conducted in the absence of any commercial or financial relationships that could be construed as a potential conflict of interest.

## Footnotes

1 http://www.peptideatlas.org/

2 http://www.peptideatlas.org/PASS/PASS01468
