# Supplementary material for: Lead Drives Complex Dynamics of a Conjugative Plasmid in a Bacterial Community
Source: Front Microbiol. 2021 May 28;12:655903. doi: 10.3389/fmicb.2021.655903 (PMC8195591; doi:10.3389/fmicb.2021.655903)
Supplement: Supplementary file 1 [file Data_Sheet_1.docx]

**Heatmap**

**Supplementary Material**

Valentine Cyriaque, Jonas Stenløkke Madsen, Laurence Fievez, Baptiste Leroy, Lars Hansen, Fabrice Bureau, Søren J. Sørensen, Ruddy Wattiez

« Lead drives complex dynamics of a conjugative plasmid in a bacterial community »

*Frontiers in Microbiology* (2021)

Functional responses of *P. putida* KT2440, *V. paradoxus* and *D. acidovorans* SPH-1 Description and heatmaps

**Variovorax paradoxus B4 *as recipient strain***

When the plasmid donor *P. putida* KT2440 was grown in the presence of *V. paradoxus* B4, proteins using Fe (II) (*e.g.* Cytochrome b) or [4 Fe-4S] (*e.g.* putative oxidoreductase or succinate dehydrogenase iron-sulfur subunit (Figure 4), Fumarate hydratase class I, C COG) as co-factor were underabundant and in some cases, replaced by an alternative class of proteins (*e.g.* Fumarate hydratase class II, Figure S8). Proteins using Mg(II) (*e.g.* isocitrate lyase, isocitrate dehydrogenase [NADP], C COG; Adenylosuccinate synthetase, formate-dependent phosphoribosylglycinamide formyltransferase, F COG) were also underabundant. Guanine deaminase (F COG), binding 1 zinc ion per subunit was underabundant at 1,5 mM of lead. Most translation, ribosomal structure and modification associated proteins (J COG) were negatively impacted by 1,5 mM of lead. Abundance of ClpV1 chaperone, peroxidases and endopeptidase (O COG) were increased with lead as well as helicases and exonucleases proteins (L COG). The membrane, especially when carrying pKJK5-*gfp*-*pbr*, was enriched in a penicillin binding protein 1B as well as toluene efflux pumps (Figure 4) and nucleotide sugar associated proteins (*mur*G associated protein) in pKJK5*-gfp* carrying cells (M COG). Membranes were also enriched in a pyoverdine ABC export system (V COG) and an efflux pump membrane transporter (P COG). Phosphate binding and transport proteins as well as efflux transporters (P COG) were superabundant at 1,5 mM of lead. The abundance of iron related proteins (*e.g.* heme oxygenase, outer membrane ferripyoverdine and heme receptors, P COG) were increased from 1 mM of lead concentration.

Impacts in *V. paradoxus* B4 protein abundances were as follow. Abundance of proteins using Zn (II) (*e.g.* Putative zinc-type alcohol dehydrogenase, C COG; ATP dependant zinc metalloprotease FtsH, O COG), Fe(II) (*e.g.* Cbb3-type cytochrome c oxidase subunit, C COG) or [4 Fe-4S] cluster (*e.g.* ferredoxin, respiratory nitrate reductase, succinate dehydrogenase, Putative formate dehydrogenase FdhB, fumarate hydratase class I, C COG) as co-factor was decreased while the abundance of the fumarate hydratase class II was increased from a concentration of 0.5 mM of lead (Figure 4). Abundance of isocitrate dehydrogenase using Mg(II) as cofactor was decreased unlike pyruvate dehydrogenase also using Mg(II) as cofactor. All translation, ribosomal structure and modification associated proteins (J COG) and most nucleotide and amino acid transport and metabolism associated proteins (E and F COGs) were negatively impacted by 1,5 mM of lead. Abundance of proteins involved in carbohydrate import was increased (*e.g.* putative sugar ABC transporters, G COG). A putative MotA/TolQ/ExbB proton channel protein (U COG), Type VI secretion system (O COG) and pilus assembly proteins (type IV system and Pil proteins, N COGs) abundances were significantly increased by 1,5 mM of lead. The glutathione-S-transferase abundance was increased at 1 mM of lead. Membrane was also strengthened by Omp proteins (M COG). Phosphate binding and transport involved proteins, catalase peroxidases, RND family efflux transporters and putative TRAP decarboxylase transporter subunits (P COG) displayed an increased abundance at 1.5 mM of lead while the abundance of TonB dependant siderophores and a ferrous iron transport protein B (P COG) were increased from a lead concentration of 1 mM.

***Delftia* *acidovorans* SPH-1 *as recipient strain***

When the *P. putida* KT2440 plasmid donor was grow in the presence of *D. acidovorans* SPH-1, proteins using [4 Fe-4S] (*e.g.* Fumarate hydratase class I, C COG; Aconitate hydratase B, C COG; or succinate dehydrogenase (Figure 4), C COG) as co-factor were underabundant at 1.5 mM of lead when pKJK5-*gfp* was used in the mating, and, in some cases, replaced by an alternative class of proteins (*e.g.* Fumarate hydratase class II, Figure S8). Proteins using Mg(II) (*e.g.* ATP synthase , malic enzyme B or isocitrate dehydrogenase , C COG; adenosyl succinate synthase, F COG) were also underabundant. Proteins using Zn (II) (*e.g.* aldolases, G COG; alcohol dehydrogenase, C COG; 30s ribosomal protein S2, J COG), Mn(II) (Poly-A polymerase I) or Co(II) (*e.g.* corrinoid adenosyl transferase, H COG) co-factors were superabundant. Lipid stock proteins (PHA synthase 2, Long-chain-fatty-acid/CoA ligase, I COG), endopeptidases, (O COG), membrane associated protein chaperone SurA (O COG), and AlgQ transcriptional regulatory protein were superabundant at 1,5 mM of lead. Membranes were enforced with superabundant lipoproteins, and nucleotide sugar associated proteins, OmpA family proteins, Penicillin binding protein1B (Figure 4) and outer-membrane efflux proteins (M COG) while porins were underabundant (M COG). Membranes were also strengthened by superabundant efflux proteins such as Cadmium translocating P-type ATPases CadII, Mg transporter, and efflux pump membrane trans (P COG). The abundance of proteins involved in phosphate entry regulation PhoB (T COG) as well as in phosphate binding and transport (P COG) was increased. Finally, proteins involved against ROS damaged (*e.g.* Glutathione S-transferase (O COG) or Flavohemoproteins (C COG) and were also superabundant at 1,5 mM while outer membrane ferripyoverdine (P COG) was over abundant at 0,5 and 1 mM.

Impacts in *D. acidovorans* SPH-1 protein abundances were as follow. Abundance of a few proteins using Fe(II) (*e.g.* Gluconate 2-dehydrogenase, C COG) or a [4 Fe-4S] cluster (*e.g.* Cytochrome c oxidase accessory protein CcoG, a 4Fe-4S ferredoxin iron-sulfur binding domain protein or a molydopterin dinucleotide-binding region including protein, C COG) as co-factor were decreased with the presence of metals. However, some of them were overabundant from a lead concentration of 0,5 mM such as nitrate reductase sub-units and an oxidoreductase FAD-binding protein (C COG). This nitrate-reductase was increased along with a nitroreductase (C COG) and D-amino-dehydrogenases (E COG). An oxidoreductase FAD/NAD(P)-binding domain protein, proteins using a [2 Fe-2S] and a FAD linked oxidase domain protein using a [4 Fe-4S] cluster as cofactor (C COGs) were superabundant at 1.5 mM of lead as well as Mg(II) cofactor dependant proteins such as ATP synthase subunits and a succinate--CoA ligase [ADP-forming] subunit beta. The membrane was strengthened by OmpA proteins and a LPS assembly protein LptD (M COG). The abundance of the chaperone HscA (O COG) was increased at 1,5 mM of lead as well as phosphatases, phosphate binding and transport associated proteins, efflux transporter RND family proteins, heavy metal efflux pumps molybdenum ABC transporters and magnesium P-type ATPases (P COG). Other efflux transporter RND family proteins, heavy metal efflux pumps as well as TonB dependant siderophores and iron permeases were overabundant from 0,5 mM of lead (P COG). Besides, a Gamma-glutamyl transferases was increased in abundance at a lead concentration of 1,5 mM (E COG). Finally, a DNA mismatch repair protein abundance was increased when lead concentration reached 1,5 mM (L COG). Notably, the TetR transcriptional regulator was increased with high lead concentration (K COG).

Following heatmaps are gathered as **Figure S7:** Heatmaps were built with centred-scaled log-2 transformed abundances of proteins classified in COGs, using Euclidean distance and average clustering.

COG clusters are defined as follow:

**Cellular processes and signalling**

**[D]** Cell cycle control, cell division, chromosome partitioning

**[M]** Cell wall/membrane/envelope biogenesis

**[N]** Cell motility

**[O]** Post-translational modification, protein turnover, and chaperones

**[T]** Signal transduction mechanisms

**[U]** Intracellular trafficking, secretion, and vesicular transport

**[V]** Defence mechanisms

**Information storage and processing**

**[J]** Translation, ribosomal structure and biogenesis

**[K]** Transcription

**[L]** Replication, recombination and repair

**Metabolism**

**[C]** Energy production and conversion

**[E]** Amino acid transport and metabolism

**[F]** Nucleotide transport and metabolism

**[G]** Carbohydrate transport and metabolism

**[H]** Coenzyme transport and metabolism

**[I]** Lipid transport and metabolism

**[P]** Inorganic ion transport and metabolism

**[Q]** Secondary metabolites biosynthesis, transport, and catabolism

**Poorly characterized**

**[S]** Function unknown


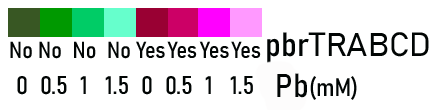

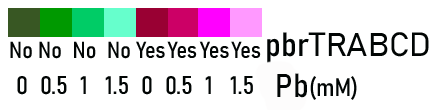

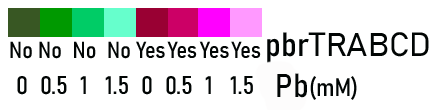

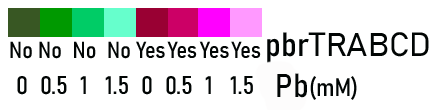


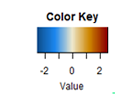

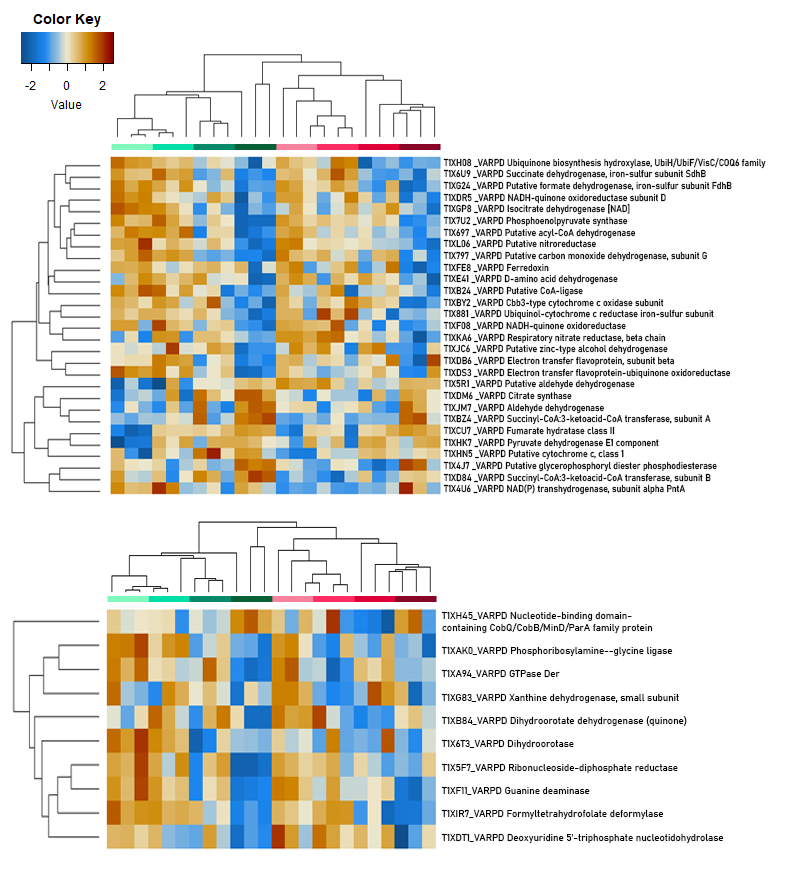


F COG

C COG

***Variovorax paradoxus* B4** and *Pseudomonas putida* KT2440


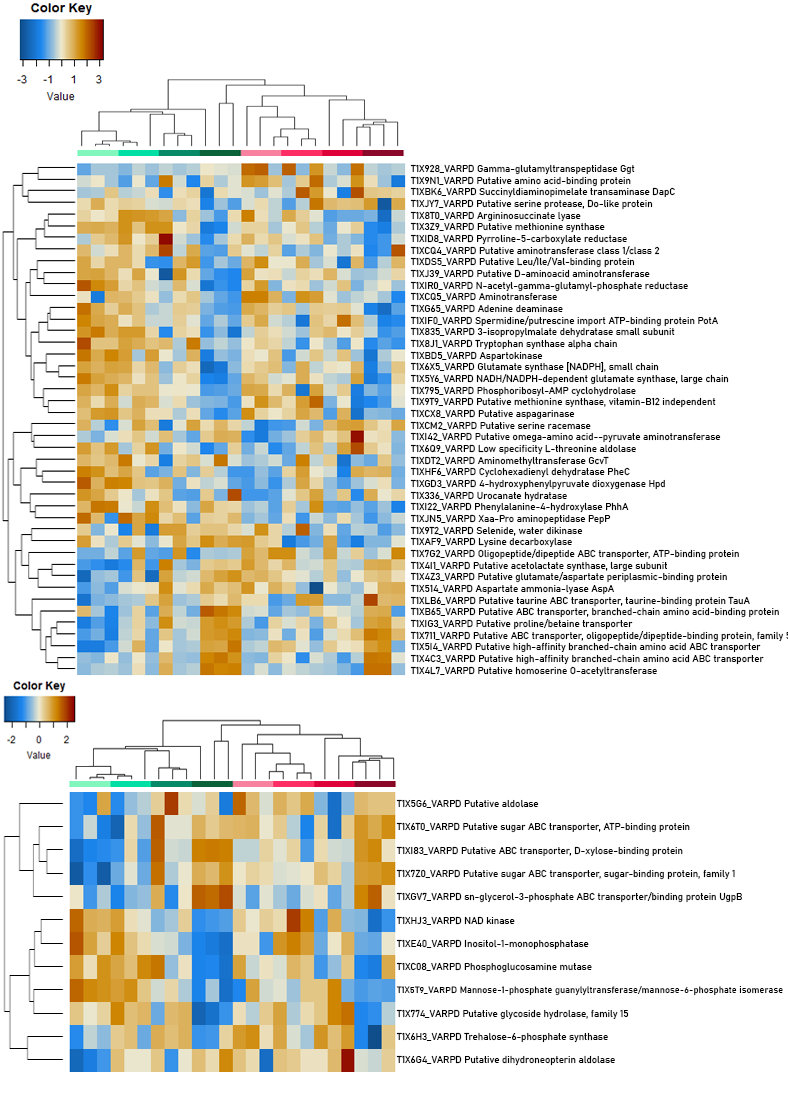


G COG

***Variovorax paradoxus* B4** and *Pseudomonas putida* KT2440

E COG


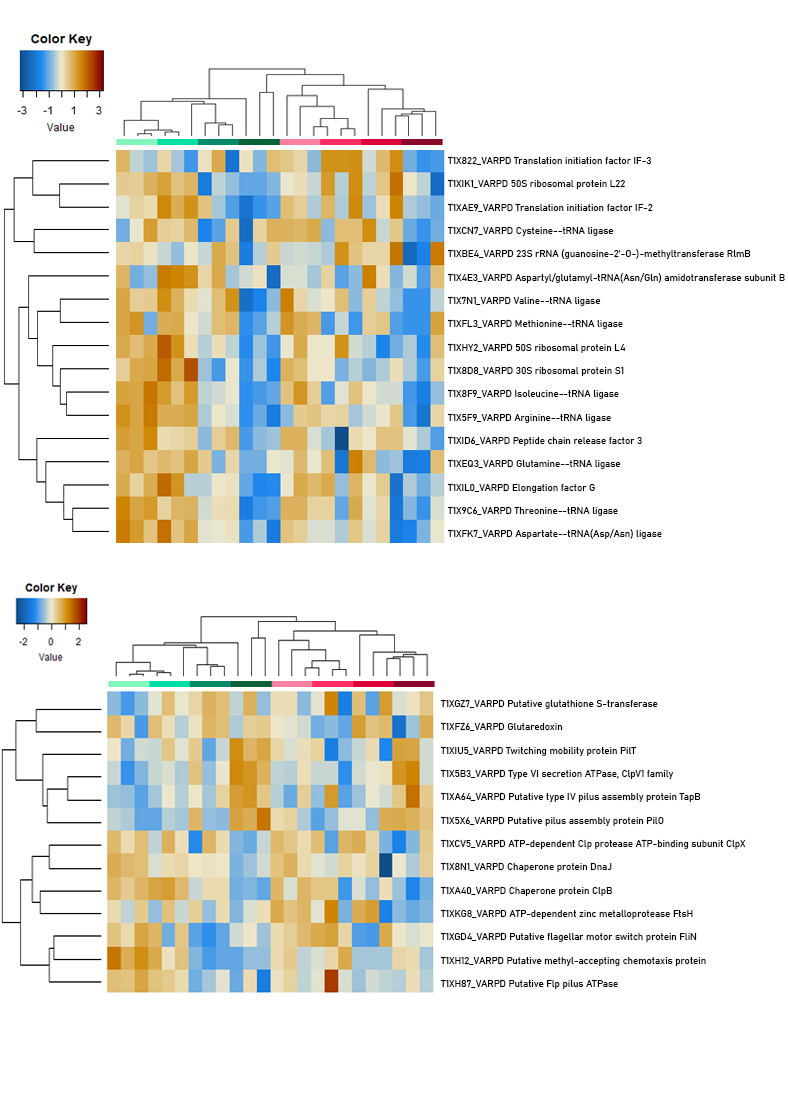


J COG

N & O COGs

***Variovorax paradoxus* B4** and *Pseudomonas putida* KT2440

***Variovorax paradoxus* B4** and *Pseudomonas putida* KT2440

M COG


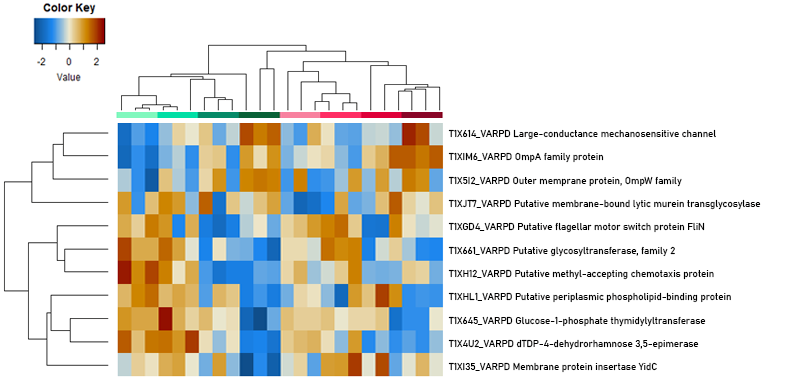


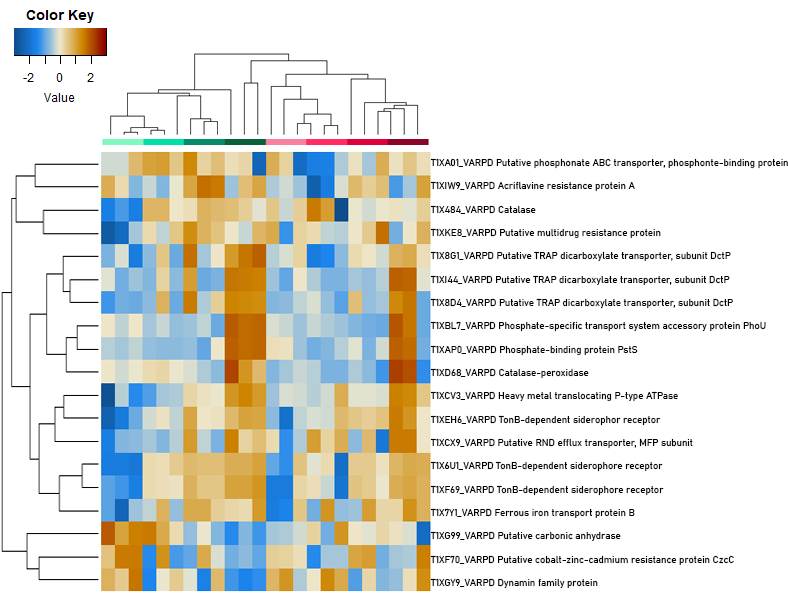


P COG


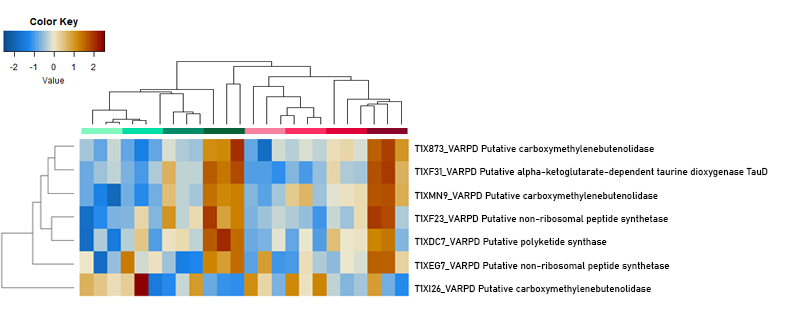


Q COG

***Variovorax paradoxus* B4** and *Pseudomonas putida* KT2440


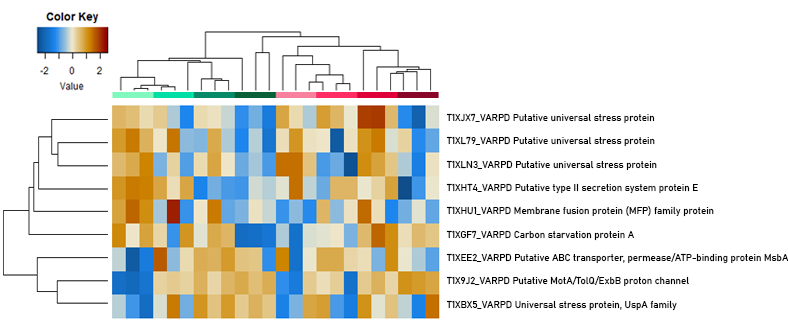


T, U & V COGs

*Variovorax paradoxus* B4 and ***Pseudomonas putida* KT2440**


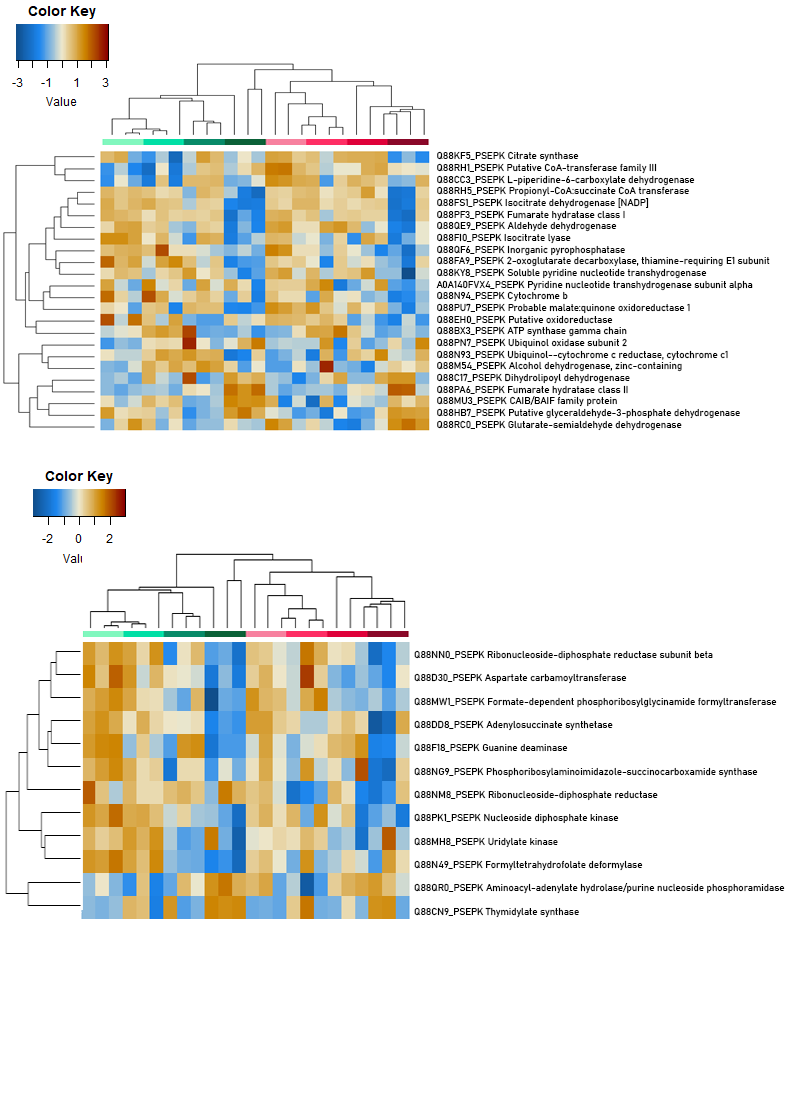


F COG

C COG

*Variovorax paradoxus* B4 and ***Pseudomonas putida* KT2440**


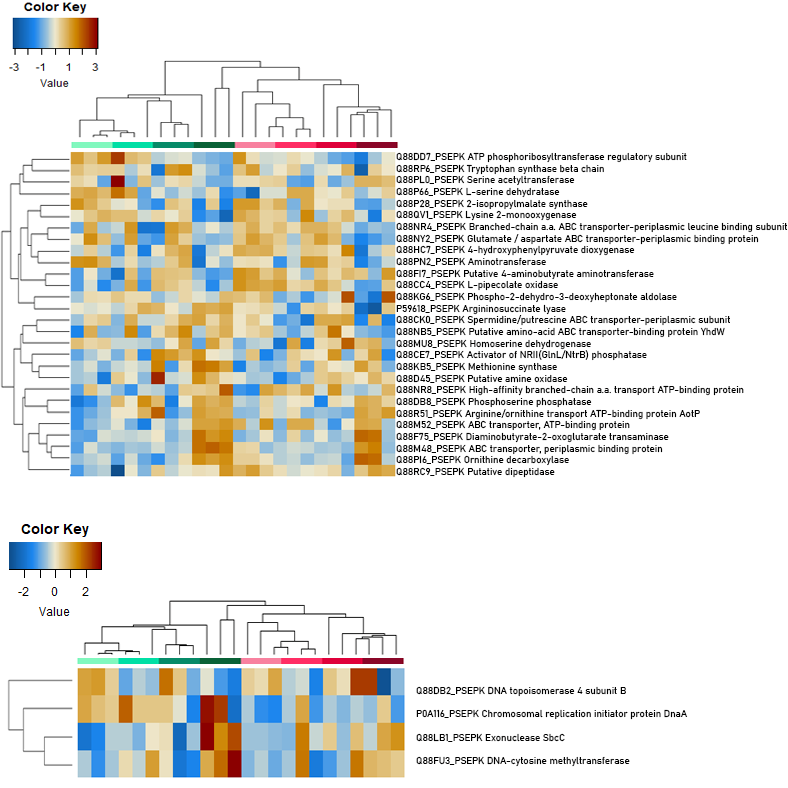


L COG

E COG

*Variovorax paradoxus* B4 and ***Pseudomonas putida* KT2440**


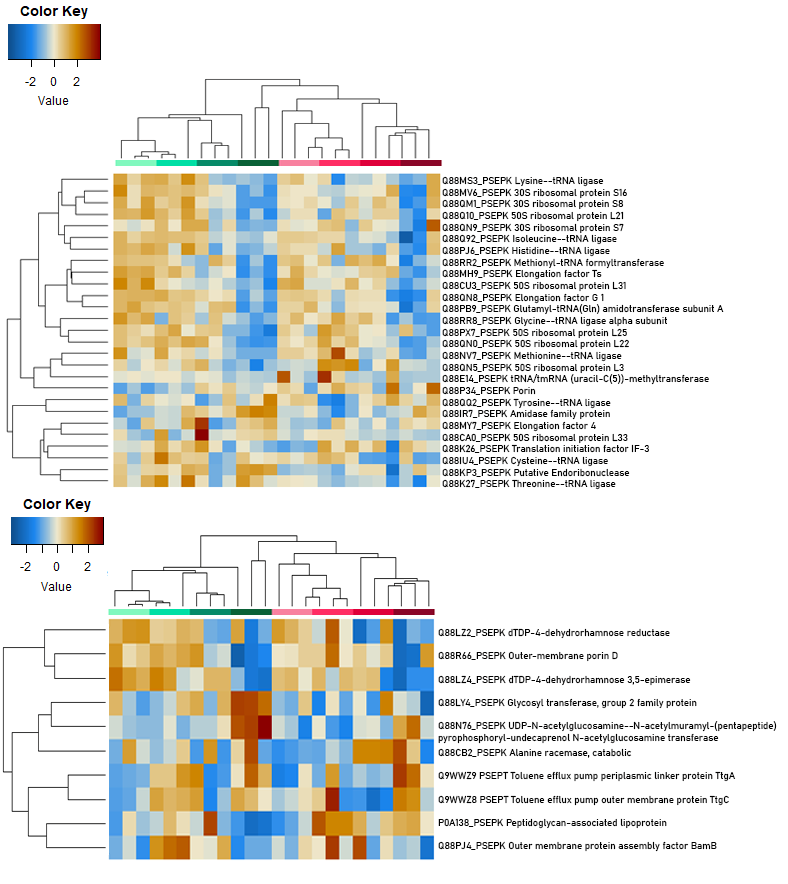


M COG

J COG


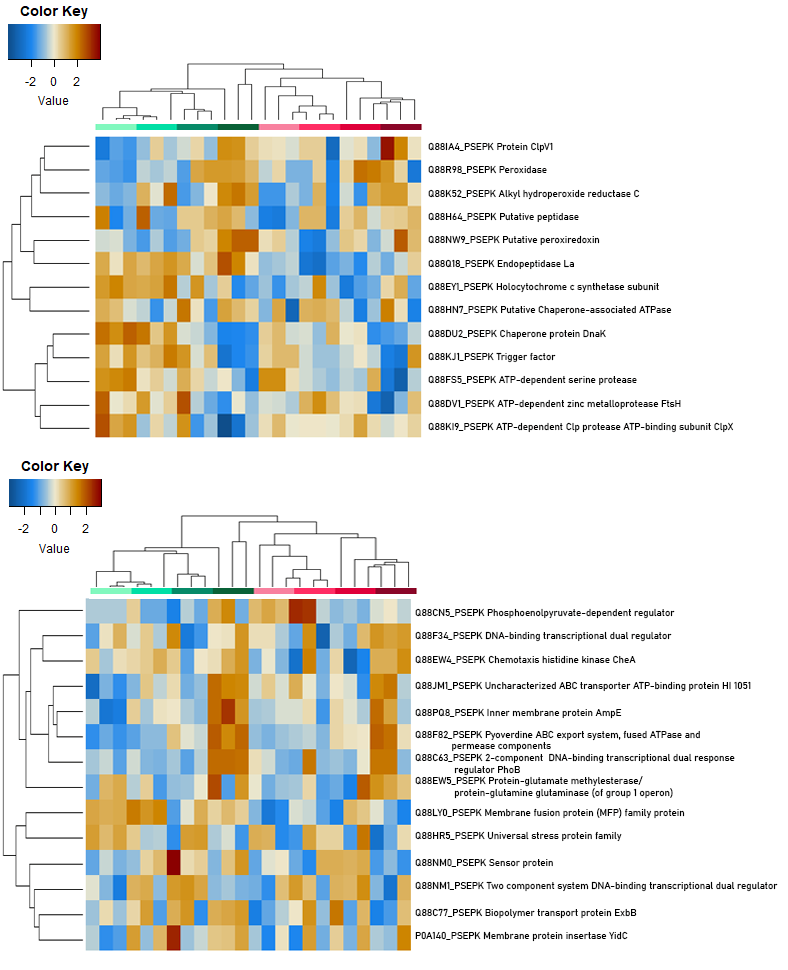


T, U & V COGs

*Variovorax paradoxus* B4 and ***Pseudomonas putida* KT2440**

O COG

*Variovorax paradoxus* B4 and ***Pseudomonas putida* KT2440**


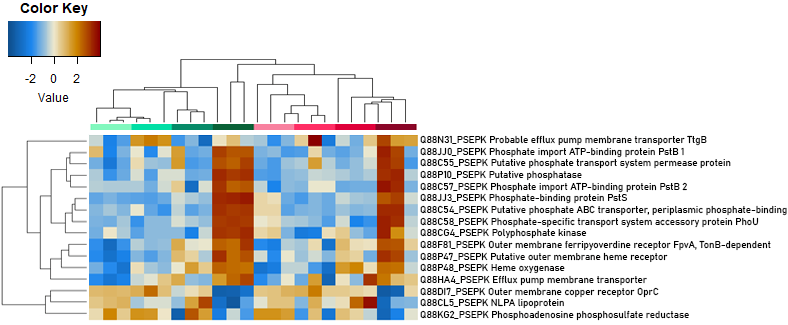


P COG

***Delftia acidovorans* SPH-1** and *Pseudomonas putida* KT2440


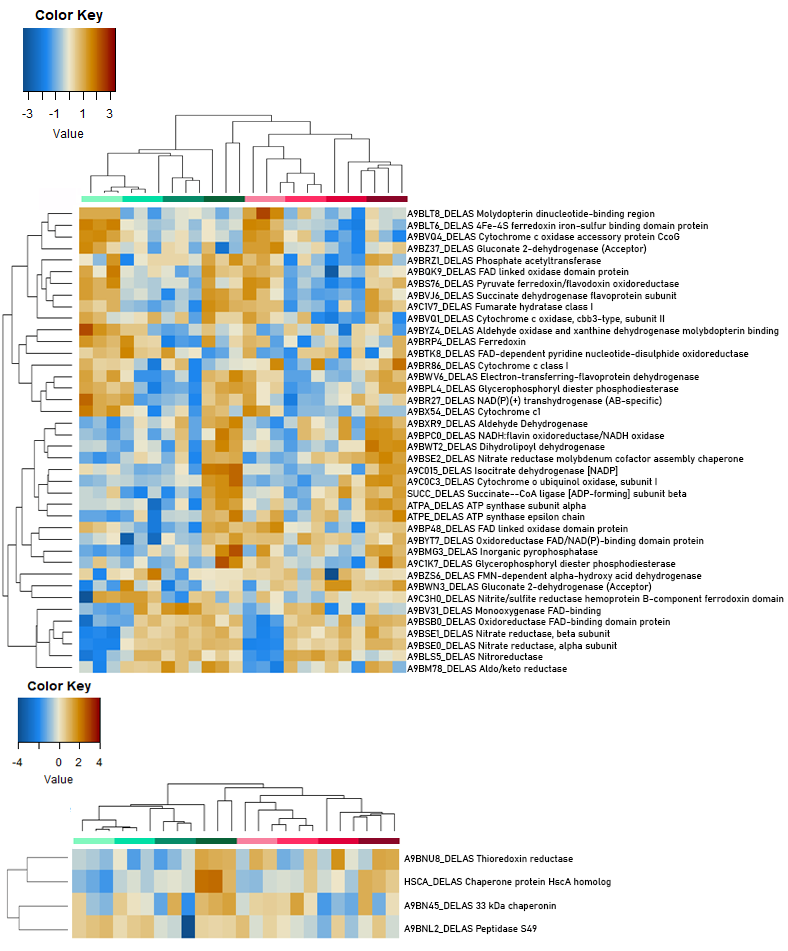


C COG

O COG

***Delftia acidovorans* SPH-1** and *Pseudomonas putida* KT2440


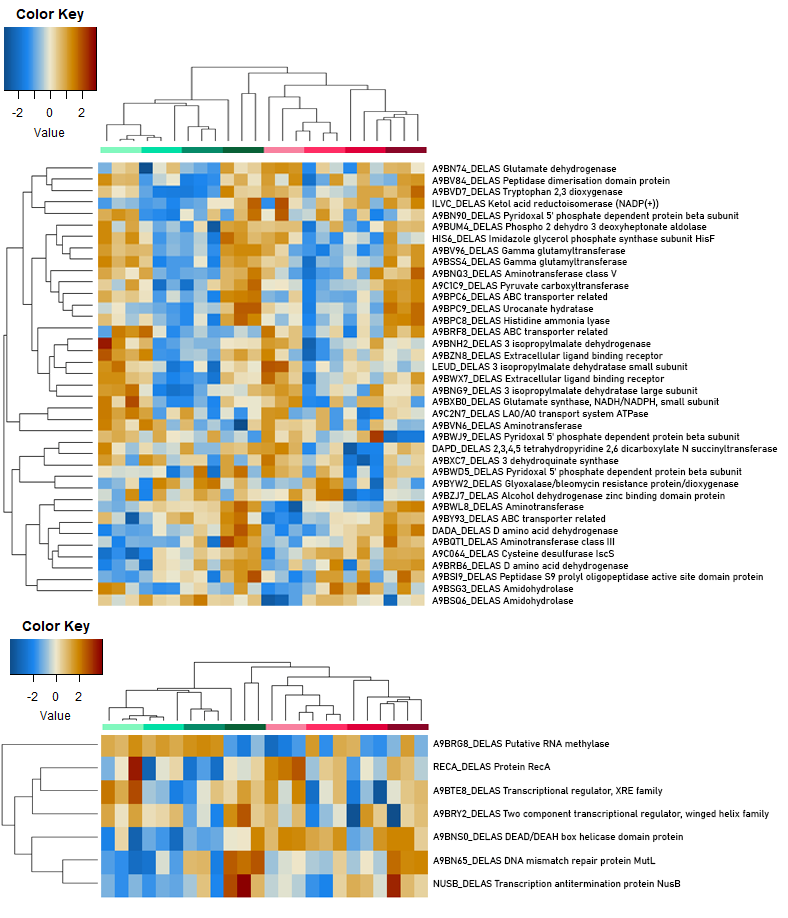


K & L COGs

E COG


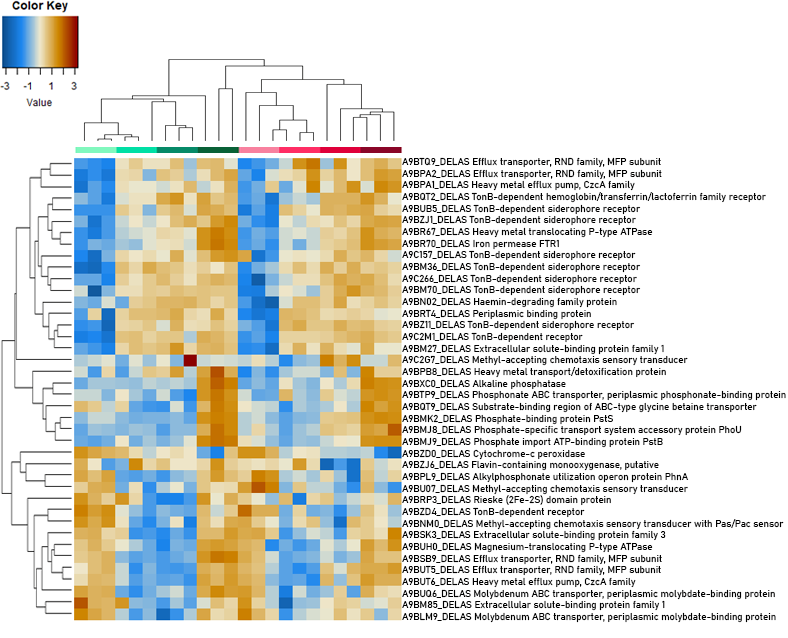


P COG

***Delftia acidovorans* SPH-1** and *Pseudomonas putida* KT2440

*Delftia acidovorans* SPH-1 and ***Pseudomonas putida* KT2440**


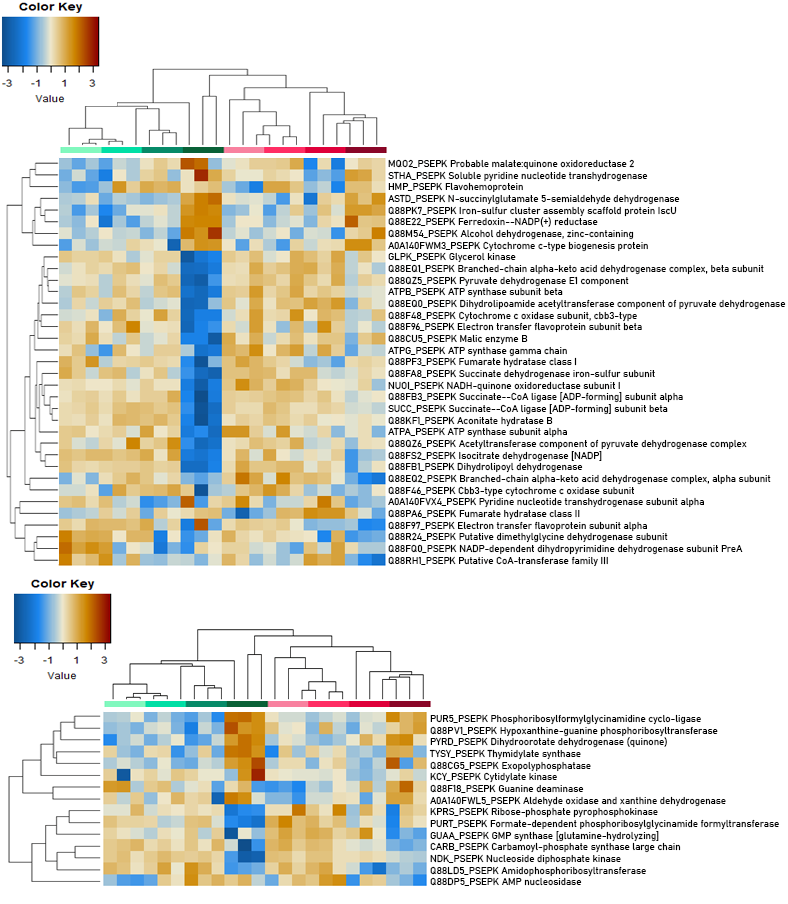


F COG

C COG

*Delftia acidovorans* SPH-1 and ***Pseudomonas putida* KT2440**


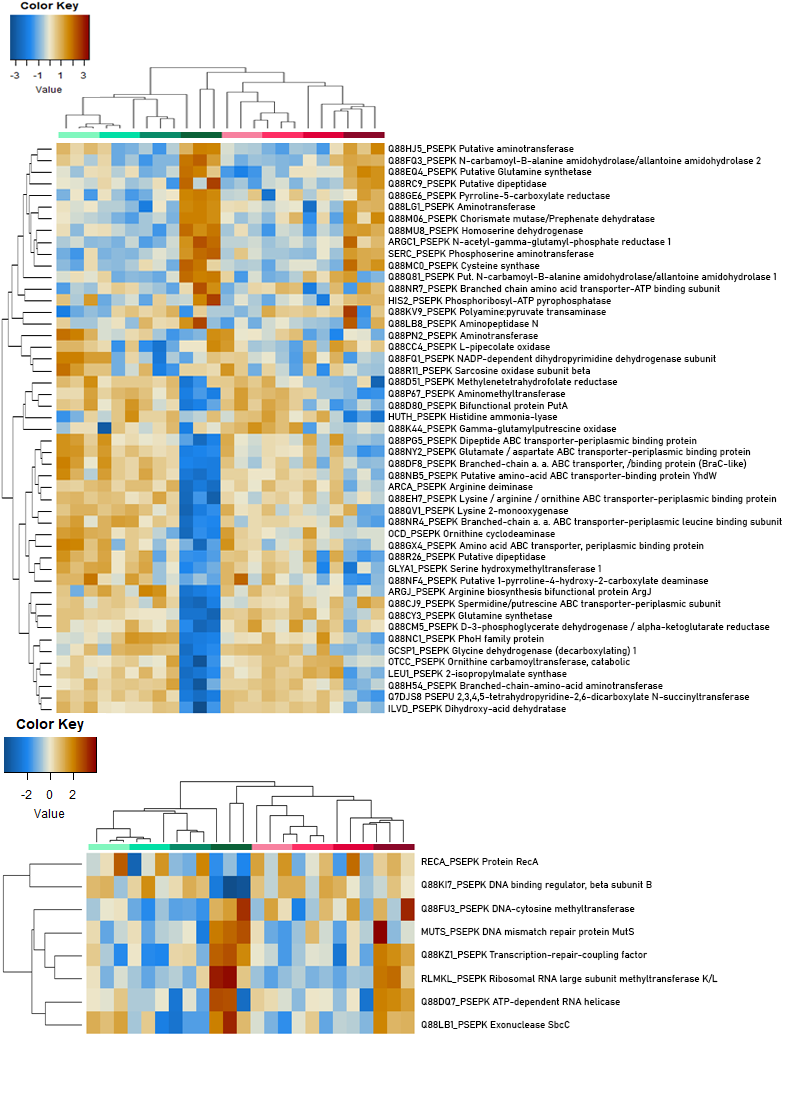


L COG

E COG

*Delftia acidovorans* SPH-1 and ***Pseudomonas putida* KT2440**


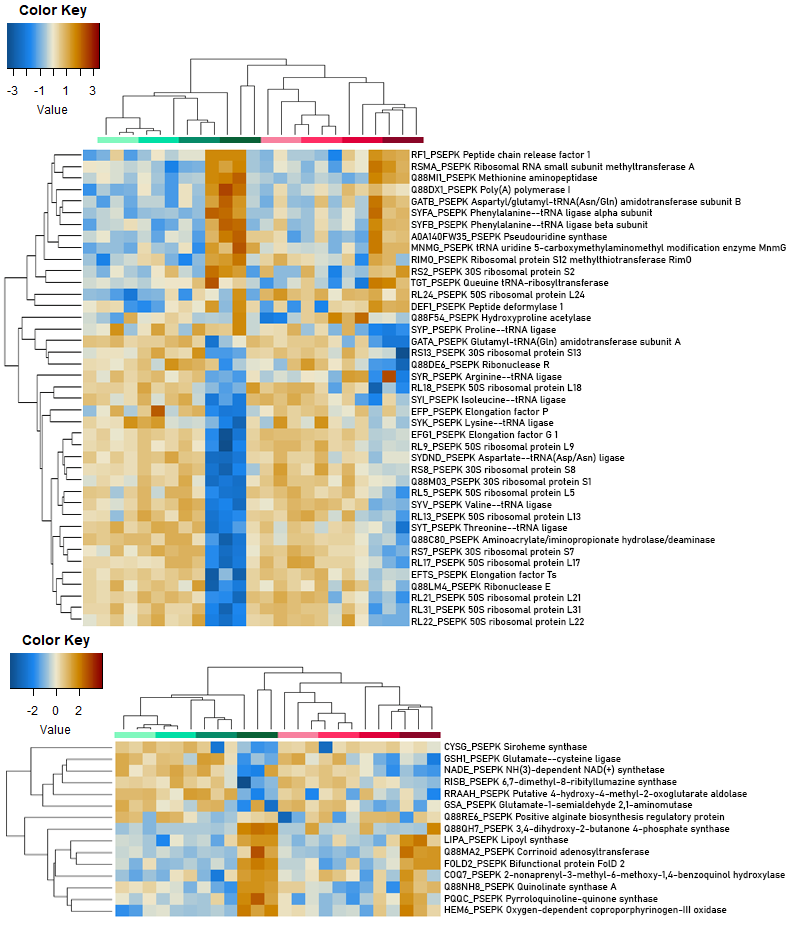


H COG

J COG

*Delftia acidovorans* SPH-1 and ***Pseudomonas putida* KT2440**


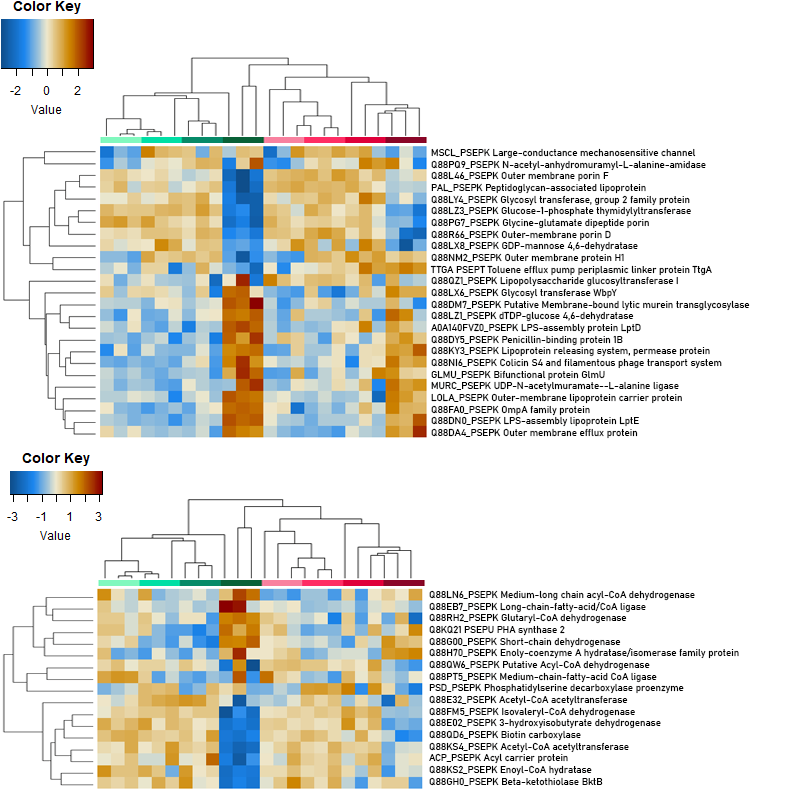


I COG

M COG

*Delftia acidovorans* SPH-1 and ***Pseudomonas putida* KT2440**


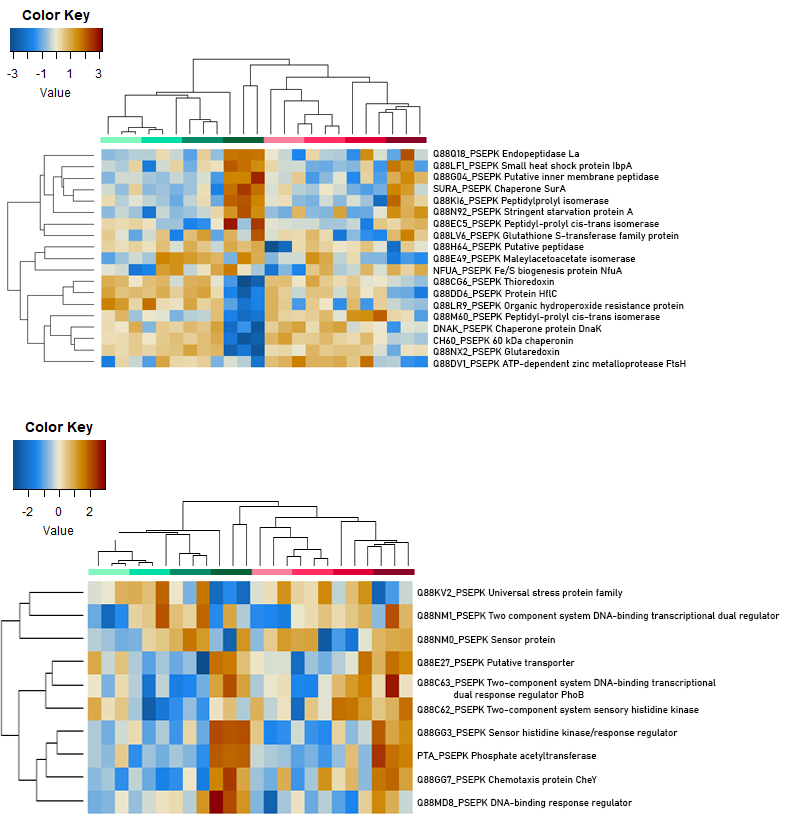


T COG

O COG

*Delftia acidovorans* SPH-1 and ***Pseudomonas putida* KT2440**


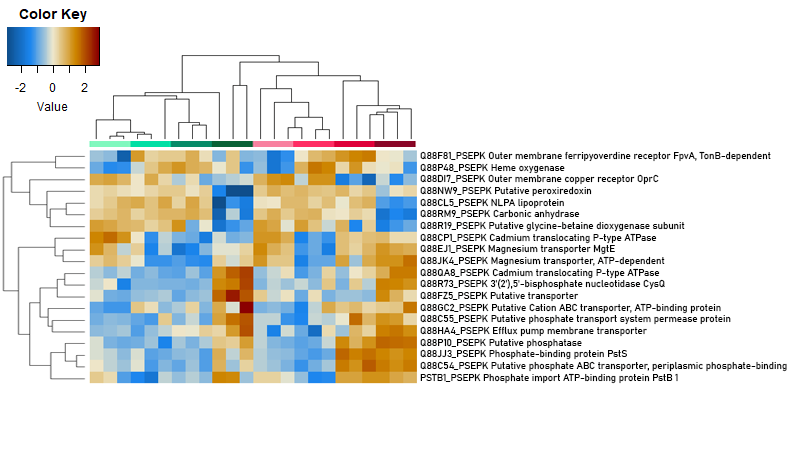


P COG
