# Supplementary material for: Lead Drives Complex Dynamics of a Conjugative Plasmid in a Bacterial Community
Source: Front Microbiol. 2021 May 28;12:655903. doi: 10.3389/fmicb.2021.655903 (PMC8195591; doi:10.3389/fmicb.2021.655903)
Supplement: Supplementary file 2 [file Data_Sheet_2.docx]

**Supplementary Figure File**

Valentine Cyriaque, Jonas Stenløkke Madsen, Laurence Fievez, Baptiste Leroy, Lars Hansen, Fabrice Bureau, Søren J. Sørensen, Ruddy Wattiez

« Lead drives complex dynamics of a conjugative plasmid in a bacterial community »

*Frontiers in Microbiology* (2021)

**
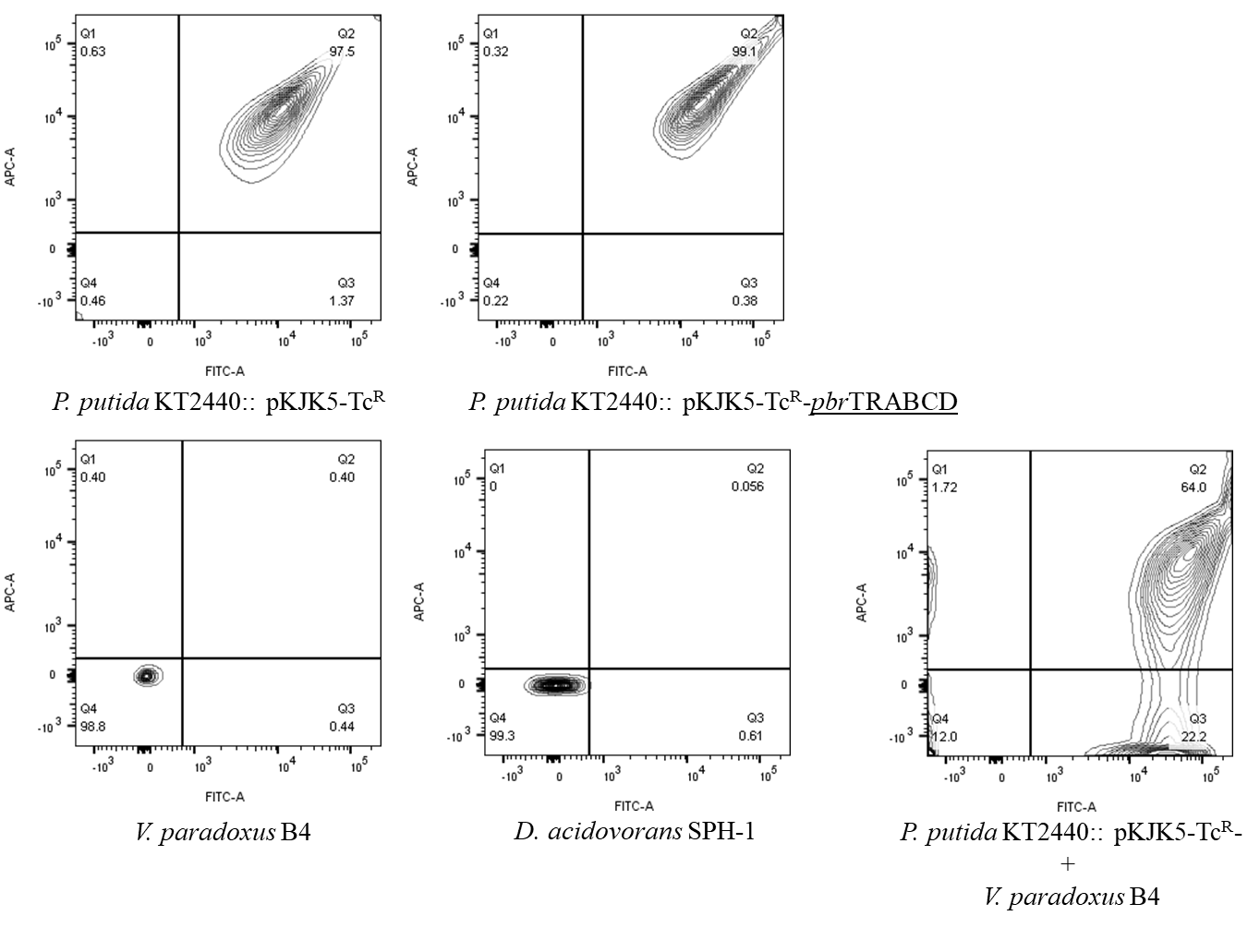
**

**Figure S1:** Example of results from flow cytometry displaying V*. paradoxus* B4, *D. acidovorans* SPH-1, *P. putida* KT2440 strains in pure-cultures and a bi-membered community gathering V*. paradoxus* B4 and *P. putida* KT2440. FACS (forward scatter 390 V, side scatter 176 V, detectors for green fluorescence associated to GFPmut fluorescence (FITC, bandpass filter 530/30 nm, 501 V) and for red fluorescence associated to AlexaFluor 647 (APC-A, bandpass filter 670/14 nm, 550 V).

**
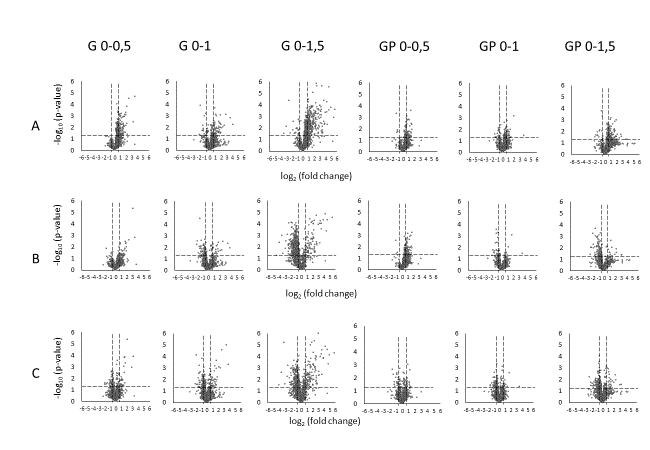
**

**
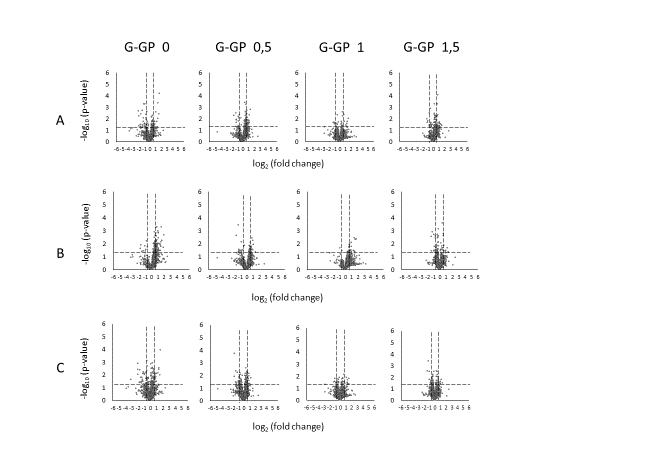
**

**Figure S2:** Patterns of differential *V. paradoxus* B4’s proteins after normalization on summed area of all proteins for each sample (A), and cell ratio in the bi-membered community obtained by flow cytometry (Cell normalized, B) or complemented with an normalization on summed area of all proteins belonging to proteins attributed to the specific strain expressions (Normalized of total proteins identified as specific strain, C). Volcano plot display the log_2_ of the fold-change ratios and the negative log_10_ of the *p*-value. Dot lines demarcates thresholds of fold changes below 0,66 (log_2_(0,66) =-0,58) and over 1,5 (log_2_(0,66) =0,58) and *p*-value below 0,05 (-log_10_(0,05) =1,30).


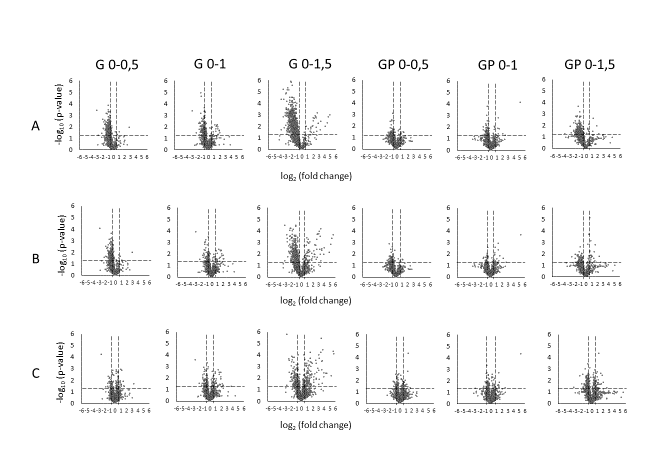


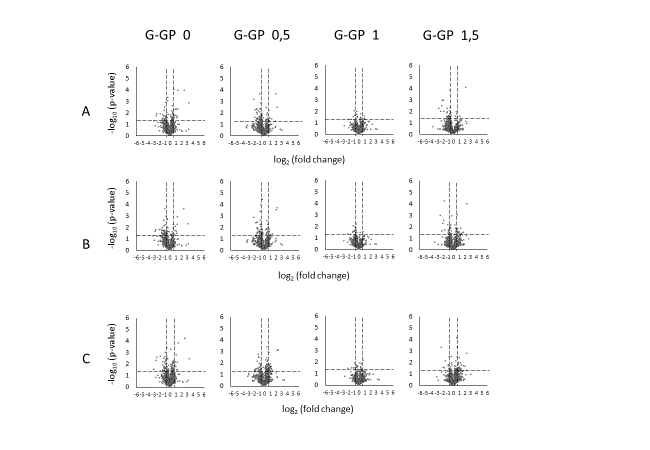


**Figure S3:** Patterns of differential *P. putida KT2440’s proteins (when associated to V. paradoxus B4)* after normalisation on summed area of all proteins for each sample (A), and cell ratio in the bi-membered community obtained by flow cytometry (Cell normalized, B) or complemented with an normalization on summed area of all proteins belonging to proteins attributed to the specific strain expressions (Normalized of total proteins identified as specific strain, C). Volcano plot display the log_2_ of the fold-change ratios and the negative log_10_ of the *p*-value. Dot lines demarcates thresholds of fold changes below 0,66 (log_2_(0,66) =-0,58) and over 1,5 (log_2_(0,66) =0,58) and *p*-value below 0,05 (-log_10_(0,05) =1,30).


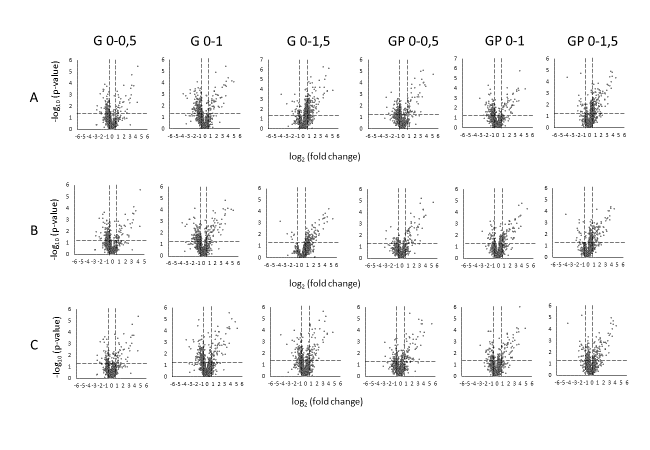


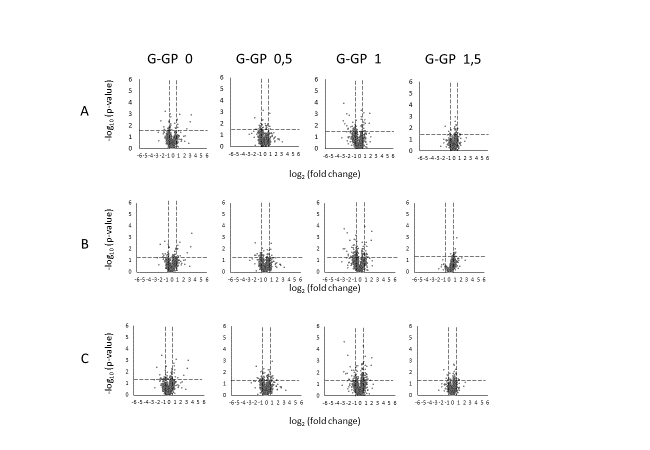


**Figure S*4*:** Patterns of differential *D.acidovorans* SPH-1’s proteins after normalisation on summed area of all proteins for each sample (A), and cell ratio in the bi-membered community obtained by flow cytometry (Cell normalized, B) or complemented with an normalization on summed area of all proteins belonging to proteins attributed to the specific strain expressions (Normalized of total proteins identified as specific strain, C). Volcano plot display the log_2_ of the fold-change ratios and the negative log_10_ of the *p*-value. Dot lines demarcates thresholds of fold changes below 0,66 (log_2_(0,66) =-0,58) and over 1,5 (log_2_(0,66) =0,58) and *p*-value below 0,05 (-log_10_(0,05) =1,30).


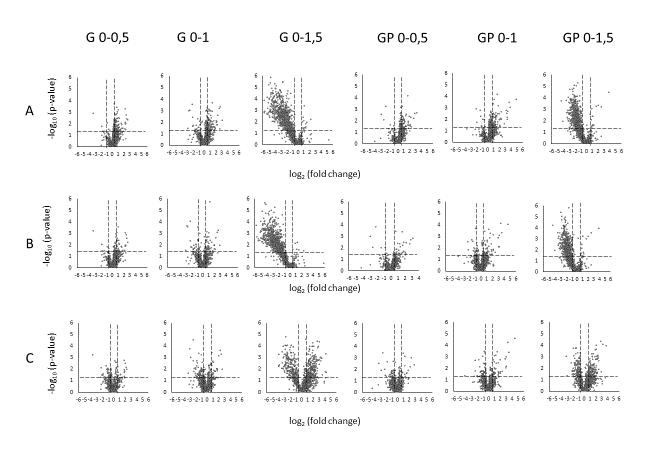


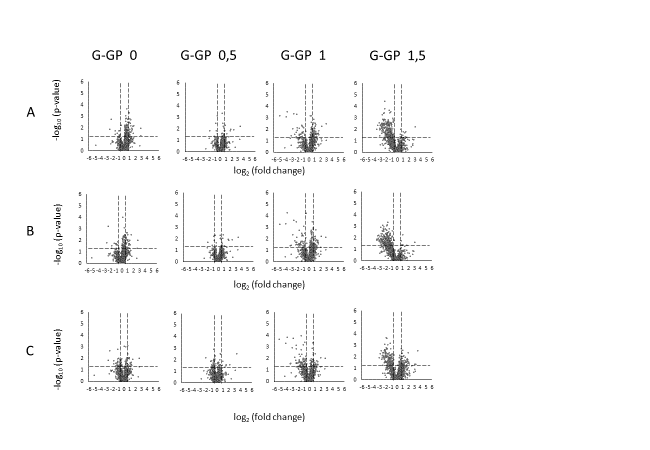


**Figure S*5*:** Patterns of differential *P. putida KT2440’s proteins (when associated to D.acidovorans SPH-1)* after normalisation on summed area of all proteins for each sample (A), and cell ratio in the bi-membered community obtained by flow cytometry (Cell normalized, B) or complemented with an normalization on summed area of all proteins belonging to proteins attributed to the specific strain expressions (Normalized of total proteins identified as specific strain, C). Volcano plot display the log_2_ of the fold-change ratios and the negative log_10_ of the *p*-value. Dot lines demarcates thresholds of fold changes below 0,66 (log_2_(0,66) =-0,58) and over 1,5 (log_2_(0,66) =0,58) and *p*-value below 0,05 (-log_10_(0,05) =1,30).

**
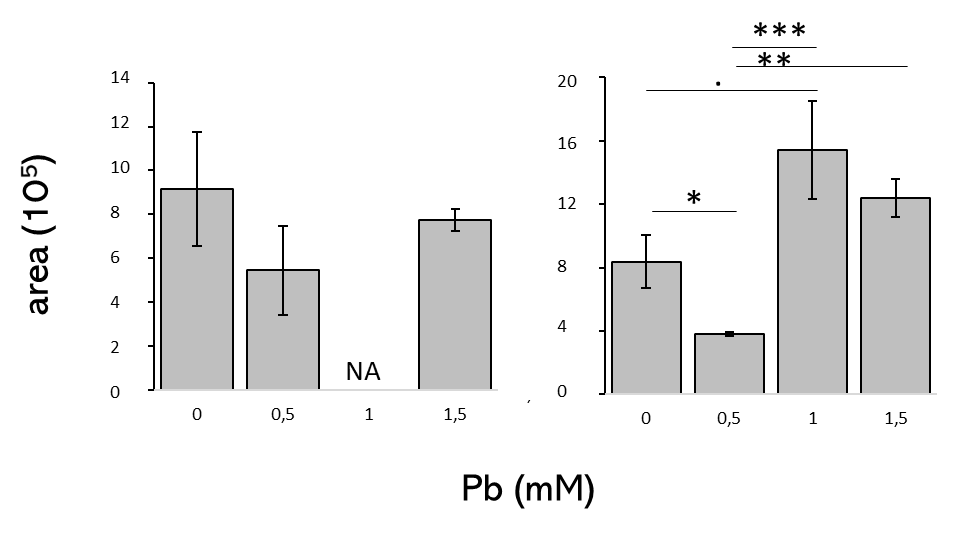
**

**Figure S6:** MRM quantification of PbrA protein in two-members communities (using either *Variovorax paradoxus* B4 (A) or *D. acidovorans* SPH-1 (B) as plasmid recipient) after normalization by the proportion of plasmid carrying cells (donors and transconjugants) obtained by flow cytometry. Significance was obtained with ANOVA followed by Tuckey test. **.** *p*-*value<0,07;* **:* p-*value<0,05; **:* p-*value<0,01; ***:* p-*value<0,001* (n=3)*.*

**Figure S7:** Supplemental heatmaps are displayed in supplemental annexed specific document (“Heatmap Supporting File”). Heatmaps were built with centred-scaled log-2 transformed abundances of proteins classified in COGs, using Euclidean distance and average clustering.
