# Supplementary material for: Lead Drives Complex Dynamics of a Conjugative Plasmid in a Bacterial Community
Source: Front Microbiol. 2021 May 28;12:655903. doi: 10.3389/fmicb.2021.655903 (PMC8195591; doi:10.3389/fmicb.2021.655903)
Supplement: Supplementary file 3 [file Data_Sheet_3.docx]

**Supplementary method**

Valentine Cyriaque, Jonas Stenløkke Madsen, Laurence Fievez, Baptiste Leroy, Lars Hansen, Fabrice Bureau, Søren J. Sørensen, Ruddy Wattiez

« Lead drives complex dynamics of a conjugative plasmid in a bacterial community »

*Frontiers in Microbiology* (2021)

**Plasmid construction and transformation**

DNA fragment including the operon *pbrTRABCD* and tetracycline resistance gene *tet^R^* (*tetC*) was inserted in the plasmid pKJK5-*gfpmut3-kan^R^*. The operon *pbrTRABCD* was amplified by PCR using genomic DNA of *Cupriavidus metallidurans* CH34 and *tet^R^* from pBR322 as templates. The two PCR product were fused by Overlap-Extension PCR (OE-PCR) DNA and plasmid extractions were carried out using DNA Extraction Genomic mini kit (A&A Biotechnology) and Plasmid Midi AX kit (A&A Biotechnology), respectively, following manufacturer instructions. PCR amplification of the *pbrTRABCD* (7091 bb) was done using primers pbrD_xbaI (5’-CATACTTCTAGACTACCTACAGGCGTAGGCAC-3’) and pbrTOLtet (5’-GGAGAACTGTGAATGCGCA**GGCGTTACACCTGGGTAGAT**-3’). PCR amplification of *tet^R^* gene (1221bp) was insured by primers Tet_SacI (5’-CGTCATGAGCTCAGGCCCTTTCGTCTTCAAGA-3’) and Tet_TOLpbr (5’-**ATCTACCCAGGTGTAACGCC**TGCGCATTCACAGTTCTCC-3’). PCRs were carried with Phusion™ Hot Start II DNA Polymerase (ThermoFisher Scientific) following the manufacturer instructions (DMSO included). Melting temperature and elongation time for *pbrTRABCD* amplification were 61°C and 3.5 minutes. Melting temperature and elongation time for *tet^R^* amplification were 63°C and 40 seconds. *pbrTRABCD* PCR product was loaded on an electrophoresis gel (10 g/L agarose; 1.5 kV, 60 minutes) and corresponding agar band was cut for DNA extraction using QIAEX II Gel Extraction Kit (Qiagen). *tet^R^* PCR products was extracted using the QIAquick PCR Purification Kit (Quiagen). OE-PCR was performed using 50 ng of both DNA fragments, Tet_SacI and pbrD_xbaI primers and the Phusion™ Hot Start II DNA Polymerase (ThermoFisher Scientific) following the manufacturer instructions (DMSO included). Melting temperature and elongation time were 62°C and 3.5 minutes.

Obtained PCR product was loaded on an electrophoresis gel (10 g/L agarose; 1.5 kV, 60 minutes) and DNA fragment (≈8 kb) was purified from the corresponding agarose band using QIAEX II Gel Extraction Kit (Qiagen). Obtained DNA fragment and the pLENTTc -Tc^S^::MCS (see following section) plasmid were cut separately using XbaI and SacI restriction enzymes (NEB; Cutsmart buffer, 90 minutes, 37°C ; stop reaction at 20 minutes, 65°C). DNA fragment (120 ng) and linearized plasmid (40 ng) were ligated together using T4 ligase (NEB) at 16°C overnight (stop reaction 65°C, 10 minutes). The obtained plasmid was introduced in Electrocomp™ GeneHogs® *E. coli* by electroporation. For that, 1 µL of DNA was gently mixed with 50 µL of electrocompetent cells and transferred into a 1 mm BioRad cuvette. Electroporation was carried out using MicroPulser™ Electroporator (BioRad) with 1.80 kV, 1 pulse. Electroporated cells were incubated for 2 hours at 30°C, 300 RPM in 500 µL SOC medium and clones were selected on tetracycline 20 µg/mL LB agar. The obtained plasmid was cut with the restriction enzyme BglII (NEB; Buffer 3, 90 minutes, 37°C; stop reaction at 20 minutes, 65°C) and the fragment of interest (Mu Ends, *pbrTRABCD* and *tet^R^* fragments, ≈8 kb) was loaded on an electrophoresis gel (10 g/L agarose; 1.5 kV, 60 minutes). The corresponding agar band was cut for DNA extraction using QIAEX II Gel Extraction Kit (Qiagen). The *pbrTRABCD*-*tet^R^* fragment was inserted into pKJK5-*gfpmut3-kan^R^* by MuA transposition using 2 µg of the plasmid and 50 ng of the BglII cut fragment. The obtained plasmid was electroporated in Electrocomp™ GeneHogs® *E. coli* as previously described. Clones were selected on tetracycline 20 µg/mL LB agar and 20 clones were grown in LB supplemented with either tetracycline (20 µg/mL) or tetracycline (20 µg/mL) and trimethoprim (50 µg/mL). One clone growing in the presence of tetracycline but not in the presence of trimethoprim was selected. Construction was checked by PCR from the purified plasmid using pbrD_xbaI and Tet_SacI primers and by sequencing adjacent regions using Mix2Seq (Eurofins) (Figure 1). The strain *Pseudomonas putida* KT2440::P*_lpp_mCherry*-*kan^R^*//pKJK5*-gfp*mut3-*pbrTRABCD-kan^R^-tet^R^* was obtained by conjugation assay between *Pseudomonas putida* KT2440::P*_lpp_mCherry*-*kan^R^* and GeneHogs® *E. coli//*pKJK5*-gfp*mut3-*pbrTRABCD-kan^R^-tet^R^*. For that, 2 mL of overnight pre-cultures of both strains were washed twice in 2 mL of hot LB (2 minutes, 7000×*g*, 30°C) and 150 µL of each were mixed in 600 µL of hot LB (30°C). The mixture was washed twice in 1 mL and resuspended in 30 µL of hot LB that was deposited on pre-heated LB-agar plate and incubated 5 hours at 37°C. The dried cell mixed was scrapped off and then resuspended in 200 µL of NaCl solution (9 g/L). Then,100 µL were diluted in 5 mL LB supplemented with tetracycline (50 µg/mL). Grown clones were isolated on LB-agar supplemented with tetracycline (50 µg/mL).


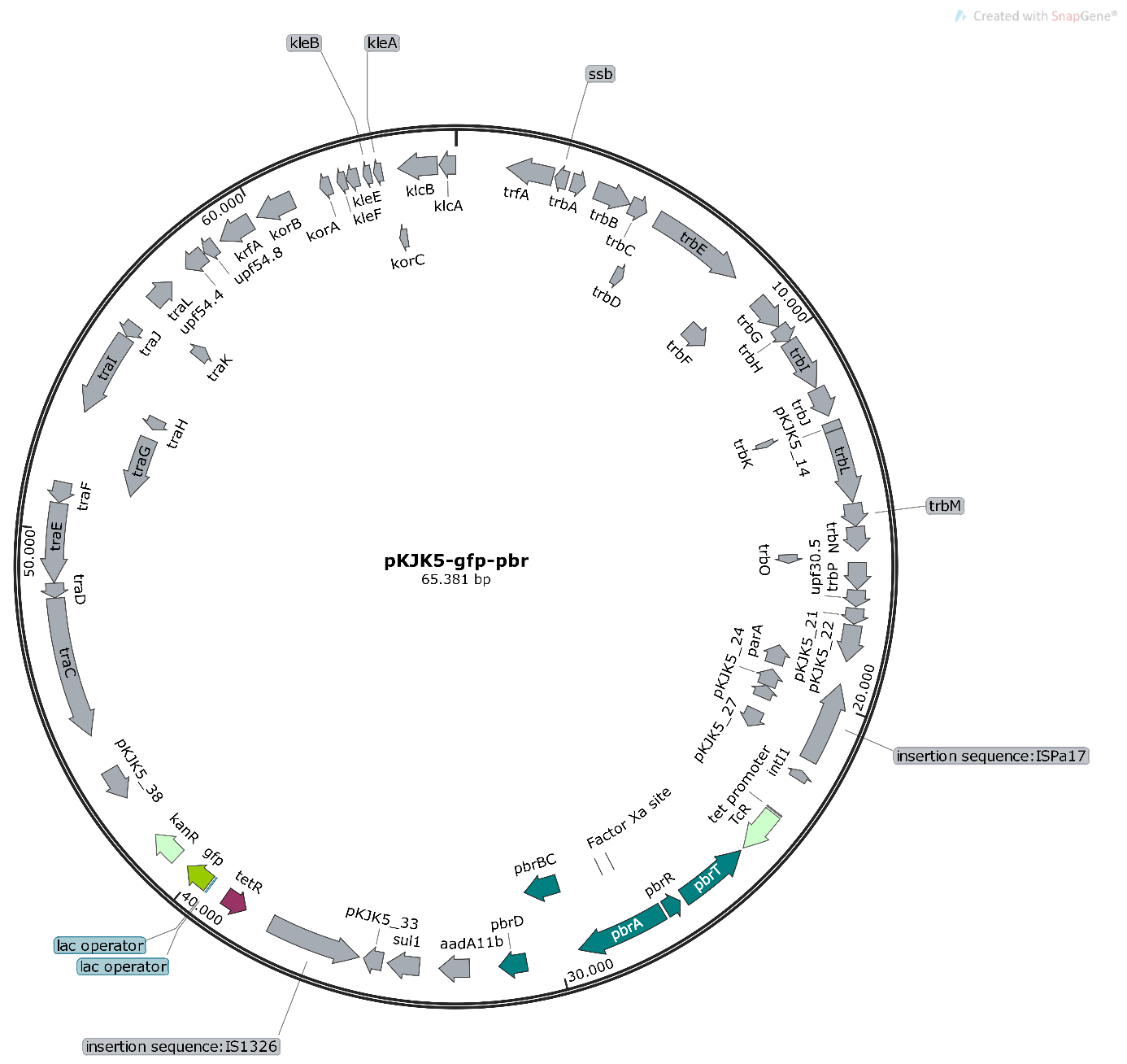


**Figure 1:** pKJK5*-gfp*mut3-*pbrTRABCD-kan^R^-tet^R^* map. Generated with SnapGene Viewer software (from Insightful Science; available at snapgene.com).

**pLENTTc -Tc^S^::MCS construction**

pLENTTc1 (Fig 1) was constructed from the pACYC177 backbone. pACYC177 was digested with *Stu*I and a 2600 bp band comprising the p15A origin of replication and the *bla* gene conferring ampicillin resistance was excised from an agarose gel and purified using QiexII (Qiagen). This was ligated to a 1599 bp PCR fragment that was generated with the primers EntklonFw 5’-GAC GTT GTA AAA CGA CGG CCA G-3’ and EntklonRev 5’-GAA ACA GCT ATG ACC ATG ATT ACG CC-3’, with pEntranceposon (tet) as template. This fragment contains the tetracycline resistance entranceposon cassette with the *tetC* gene and surrounding *Bgl*II sites flanking the MuA ends. The ligation mix was electroporated into *E. coli* Genehogs and transformants were selected on LB agar with ampicillin (100µg/ml) and tetracycline (10µg/ml). The pLENTTc1 construct was then verified using *Bgl*II restriction enzyme digests followed by electrophoresis and by sanger sequencing out from the entranceposon part with two different primers EnttetFW (5’- GTC AAA CAT GAG AAG GAT CCG-3’) and SeqA (5’- ATC AGC GGC CGC GAT C-3’). From the sequencing reaction the orientation of the insert could be established. pLENTTc and pUCP22not plasmids were cut separately using HindIII and NotI HF (pLENTTc) or NotI HF (pUCP22not) restriction enzymes (NEB; Cutsmart buffer, 90 minutes, 37°C ; stop reaction at 20 minutes, 65°C). pLENTTc1 and MCS obtained fragments were ligated using T4 ligase (NEB) at 16°C overnight (stop reaction 65°C, 10 minutes). The obtained plasmid was introduced in Electrocomp™ GeneHogs® *E. coli* by electroporation as described in the main text. The sequence of the final pLENTTc vector was verified by whole genomes sequencing using the Illumina NextSeq platform (Figure 2).


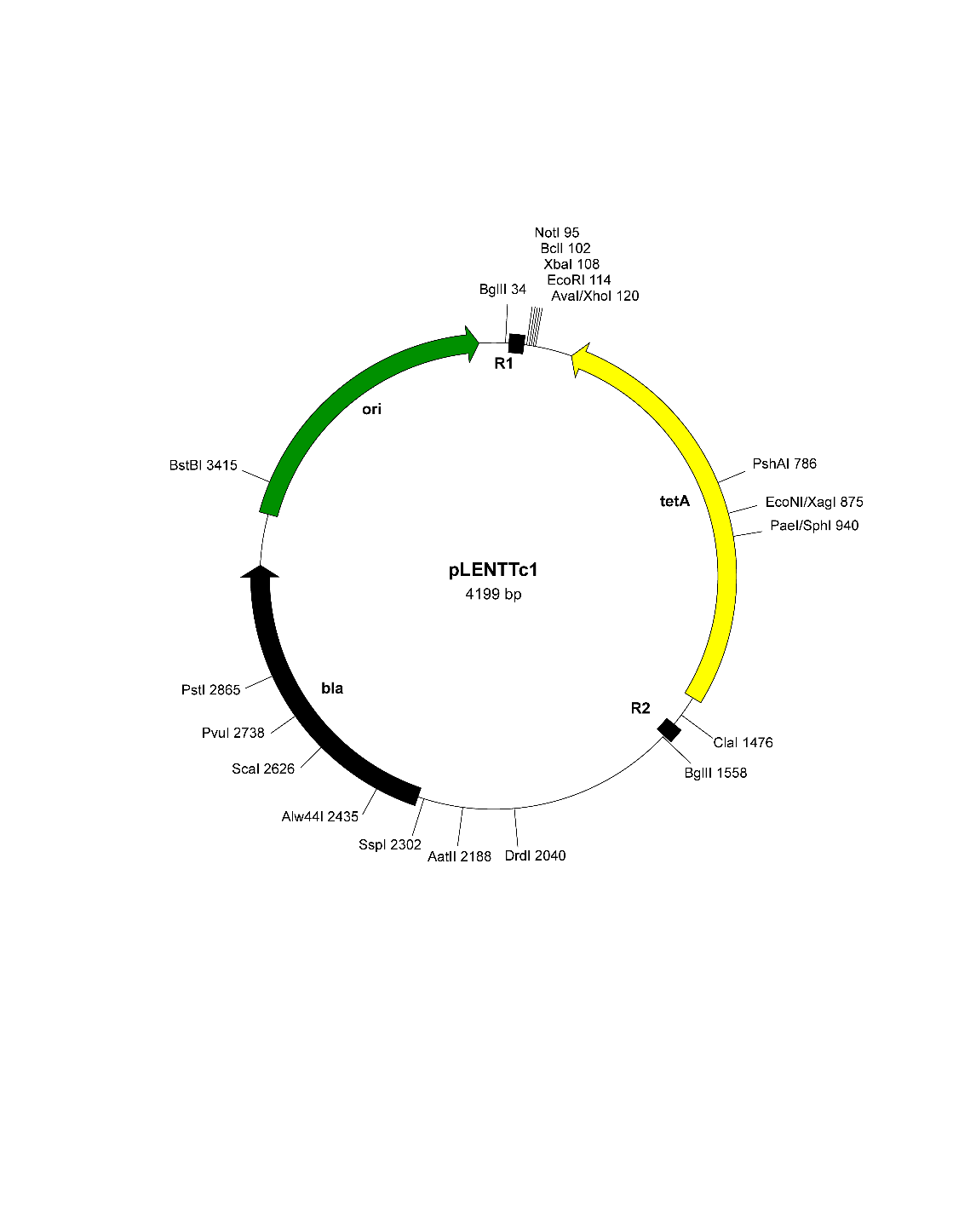


**Figure 2 :** pLENTTc map

***V. paradoxus* B4 and *D. acidovorans* SPH-1 transformation with pKJK5 plasmids for burden assay**

From overnight pre-cultures of each plasmid recipient and donor cell cultures (GeneHogs® *E. coli-* pKJK5-*gfp* or GeneHogs® *E. coli-* pKJK5*-gfp*-*pbr*), 2 mL were washed twice in 2 mL of hot LB (2 minutes, 7000×*g*, 37°C) and 150 µL were mixed in 600 µL of hot LB (37°C). The mix was washed twice in 1 mL and resuspended in 30 µL of hot LB that was dropped on pre-heated LB-agar plate incubated overnight at 37°C. The dried cell mix was scrapped, resuspended in 200 µL of NaCl (9g/L) whose 100 µL were spread and incubated overnight either on LB plate supplemented with ampicillin (100 µg/mL) and tetracycline (20 µg/mL) for *D. acidovorans* selection or on 457- agar medium supplemented with tetracycline (20 µg/mL) for *V. paradoxus* selection*.*

**SWATH metaproteomic analysis**

Tryptic peptides were separated on a C18 column (Acclaim PepMap100, 3 μm, 150 μm × 25 cm, Dionex) with a linear acetonitrile gradient (5 to 35% of acetonitrile (v/v), 0,1% FA, 300 nl.min-1, 120 min) in water containing 0.1% (v/v) formic acid. MS survey scans (m/z 400-1250, 100 ms accumulation time) were succeeded by 50 SWATH acquisition overlapping windows covering the precursor m/z range. Ion collision induced dissociation were carried on using rolling collision energy, and fragment ion were accumulated for 95 ms in high sensitivity mode. SWATH technology identifies obtained spectra by comparing them to a referential spectral library built by Data-Dependent Acquisition (DDA). To build the library, proteins were extracted and digested using the PreOmics kit (PreOmics GmbH, Germany) following the manufacturer instructions, from monoculture of each used strain and cocultures (Table S1) at lead concentration of 0 mM and 1 mM. Parameters used to acquire the DDA spectra were as follow: MS scan (m/z 400-1500, 500 ms accumulation time) followed by 50 MS/MS scans (m/z 100-1800, 50 ms accumulation time, intensity threshold at 200 c.p.s). AB Sciex ProteinPilot™ 4.5 software was used to process the DDA mass spectrometry data. Spectra identification was performed by searching against the corresponding strain UniProt entries with parameters including carbamidomethyl cysteine, oxidized methionine, all biological modifications, amino acid substitutions and missed cleavage site. The final SWATH reference spectral library referred to the proteins identified at a false discovery rate below 1%.
