# Supplementary material for: Lead Drives Complex Dynamics of a Conjugative Plasmid in a Bacterial Community
Source: Front Microbiol. 2021 May 28;12:655903. doi: 10.3389/fmicb.2021.655903 (PMC8195591; doi:10.3389/fmicb.2021.655903)
Supplement: Supplementary file 4 [file Data_Sheet_4.docx]

**Supplementary Table File**

Valentine Cyriaque, Jonas Stenløkke Madsen, Laurence Fievez, Baptiste Leroy, Lars Hansen, Fabrice Bureau, Søren J. Sørensen, Ruddy Wattiez

« Lead drives complex dynamics of a conjugative plasmid in a bacterial community »

*Frontiers in Microbiology* (2021)

**Table S1:** Overview of strain characteristics

|  | *Pseudomonas putida* | *Variovorax paradoxus* | *Delftia acidovorans* |
| --- | --- | --- | --- |
| Cell size | 0.5-0.6 x 1.4-1.7 µM^a^ | 0.5-0.7 x 1.2-3.0 µM^b^ | 0.5-0.7 x 1.7-2.5 µM^c^ |
| MIC (3-times diluted LB) (mM) | 2 | 2 | 2 |
| OD max [Pb] =0 | 1.30±0.03 | 1.36 ± 0.02 | 1.11±0.02 |
| OD max [Pb] =1mM | 0.7 | 0.9 | 0.9 |
| [Prot] (µg/µL of culture) [Pb] =0 | 0.69 | 0.7 | 0.41 |
| [Prot] (µg/µL of culture) [Pb] =1mM | 0.34 | 0.35 | 0.41 |
| Genome size (Mb) (NCBI) | 6.18 | 6.55 | 6.77 |
| #proteins (uniprot) | 5947 | 6764 | 5970 |
|  |  | a | Nikolajeva et al. (2012) |
|  |  | b | Satola et al. (2013) |
|  |  | c | Loo et al. (2007) |

**Table S2**: Cell composition of start cultures for burden assay and conjugation assay experiments.

|  |  | **Strain name** | **Plasmid** | **Starting number of cells** |
| --- | --- | --- | --- | --- |
| Burden assay | Strain 1 | *P. putida* KT2440:: Plpp-mCherry-Km^R^ | - | 6,000 |
|  |  |  |  |  |
|  | Strain 1 | *P. putida* KT2440:: Plpp-mCherry-Km^R^ | *pKJK5*-gfpmut3*-Km^R^-Tet^R^* | 6,000 |
|  |  |  |  |  |
|  | Strain 1 | *P. putida* KT2440:: Plpp-mCherry-Km^R^ | *pKJK5-*gfpmut3-*pbr*TRABCD*-Km^R^-Tet^R^* | 6,000 |
|  |  |  |  |  |
|  | Strain 1 | *V. paradoxus* B4 | - | 60,000 |
|  |  |  |  |  |
|  | Strain 1 | *V. paradoxus* B4 | *pKJK5*-gfpmut3*-Km^R^-Tet^R^* | 60,000 |
|  |  |  |  |  |
|  | Strain 1 | *V. paradoxus* B4 | *pKJK5-*gfpmut3-*pbr*TRABCD*-Km^R^-Tet^R^* | 60,000 |
|  |  |  |  |  |
|  | Strain 1 | *D. acidovorans* SPH-1 | - | 60,000 |
|  |  |  |  |  |
|  | Strain 1 | *D. acidovorans* SPH-1 | *pKJK5*-gfpmut3*-Km^R^-Tet^R^* | 60,000 |
|  |  |  |  |  |
|  | Strain 1 | *D. acidovorans* SPH-1 | *pKJK5-*gfpmut3-*pbr*TRABCD*-Km^R^-Tet^R^* | 60,000 |
|  |  |  |  |  |
| Conjugation assay | Strain 1 | P. putida KT2440:: Plpp-mCherry-Km^R^ | *pKJK5*-gfpmut3*-Km^R^-Tet^R^* | 6,000 |
|  | Strain 2 | *V. paradoxus* B4 | - | 60,000 |
|  |  |  |  |  |
|  | Strain 1 | P. putida KT2440:: Plpp-mCherry-Km^R^ | *pKJK5-*gfpmut3-*pbr*TRABCD*-Km^R^-Tet^R^* | 6,000 |
|  | Strain 2 | *V. paradoxus* B4 | - | 60,000 |
|  |  |  |  |  |
|  | Strain 1 | P. putida KT2440:: Plpp-mCherry-Km^R^ | *pKJK5*-gfpmut3*-Km^R^-Tet^R^* | 6,000 |
|  | Strain 2 | *D. acidovorans* SPH-1 | - | 60,000 |
|  |  |  |  |  |
|  | Strain 1 | P. putida KT2440:: Plpp-mCherry-Km^R^ | *pKJK5-*gfpmut3-*pbr*TRABCD*-Km^R^-Tet^R^* | 6,000 |
|  | Strain 2 | *D. acidovorans* SPH-1 | - | 60,000 |
|  |  |  |  |  |

**Table S3:** Growth rate of Variovorax paradoxus B4, Delftia acidovorans SPH-1 and Pseudomonas putida KT2440 as plasmid-free cells (0) or hosting pKJK5-*gfp*  or pKJK5-*gfp-pbr* plasmids.

**Table S4:** Strain proportions as recorded by flow cytometry and summed area of all proteins assigned to corresponding strain.

**Table S5:** Selected 4 peptides and corresponding transitions for quantification, after transition optimisation and interference removal.

| **Peptide sequence** | **Precursor and charge** | **Ion fragment*** |
| --- | --- | --- |
| **NPEPSTVGAGLK** | 585,311677+2 | 829,477794+[y9] |
|  | 585,311677+2 | 445,276909+[y5] |
|  | 585,311677+2 | 194,631361++[y4] |
|  | 585,311677+2 | 212,102967+[b2] |
|  | 585,311677+2 | 341,14556+[b3] |
| **IALDGQVIEGR** | 585,827494+2 | 986,526535+[y9] |
|  | 585,827494+2 | 873,442471+[y8] |
|  | 585,827494+2 | 758,415528+[y7] |
|  | 585,827494+2 | 474,267073+[y4] |
| **VTAAANASTLAR** | 573,317294+2 | 874,474105+[y9] |
|  | 573,317294+2 | 803,436992+[y8] |
|  | 573,317294+2 | 732,399878+[y7] |
|  | 573,317294+2 | 618,35695+[y6] |
| **QAIADLHTLGVK** | 422,578588+3 | 654,393336+[y6] |
|  | 422,578588+3 | 517,334424+[y5] |
|  | 422,578588+3 | 533,816398++[y10] |
|  | 422,578588+3 | 477,274366++[y9] |
|  | 422,578588+3 | 441,755809++[y8] |
|  |  |  |
| *Used fragment were selected according to their intensity and non-interference with the precursor | | |

**Table S6:** Number of identified proteins (confidence 99%) found in the mono-culture coding for potential metal-resistance involved proteins of either *Pseudomonas putida* KT2400, *Variovorax paradoxus* B4, *Delftia acidovorans* SPH-1 used for building the protein library for SWATH proteomics in duplicate. See Supplemental Table S7.

| ***Pseudomonas putida* KT2240** |  |  | ***pKJK5-GFP*** | | | | ***pKJK5-GFP-pbr*** | | | |
| --- | --- | --- | --- | --- | --- | --- | --- | --- | --- | --- |
|  |  |  | Pb (mM) | | | | | | | |
|  | **code** | **function** | **0** | **0** | **1** | **1** | **0** | **0** | **1** | **1** |
|  |  |  |  |  |  |  |  |  |  |  |
| **Chaperone** | Q88N55 | 60 kDa chaperonin | 65 | 57 | 50 | 52 | 42 | 48 | 52 | 50 |
|  | Q88Q71 | Chaperone protein ClpB | 33 | 39 | 31 | 39 | 35 | 39 | 37 | 34 |
|  | Q88DU3 | Chaperone protein DnaJ | 6 | 4 | 7 | 9 | 7 | 5 | 6 | 7 |
|  | Q88DU2 | Chaperone protein DnaK | 41 | 45 | 37 | 44 | 34 | 35 | 32 | 32 |
|  | Q88PK4 | Chaperone protein HscA homolog | 10 | 8 | 5 | 5 | 10 | 6 | 4 | 2 |
|  | Q88FB9 | Chaperone protein HtpG | 28 | 21 | 22 | 24 | 24 | 19 | 19 | 17 |
|  | Q88QT4 | Chaperone SurA | 9 | 8 | 6 | 7 | 9 | 6 | 4 | 7 |
|  | Q88HN7 | Putative Chaperone-associated ATPase | 9 | 14 | 12 | 14 | 5 | 7 | 16 | 9 |
|  | Q88KV7 | RNA chaperone ProQ | 5 | 5 | 5 | 7 | 4 | 5 | 5 | 7 |
|  | Q88HN7 | Putative Chaperone-associated ATPase | 9 | 14 | 12 | 14 | 5 | 7 | 16 | 9 |
|  | Q88KV7 | RNA chaperone ProQ | 5 | 5 | 5 | 7 | 4 | 5 | 5 | 7 |
|  |  |  |  |  |  |  |  |  |  |  |
|  | Q9KJC2 | Antibiotic efflux pump membrane transporter ArpB | 9 | 6 | 7 | 10 | 7 | 5 | 11 | 11 |
| **Efflux** | Q9KJC1 | Antibiotic efflux pump outer membrane protein ArpC | 11 | 14 | 16 | 14 | 16 | 17 | 20 | 15 |
|  | Q9KJC3 | Antibiotic efflux pump periplasmic linker protein ArpA | 10 | 4 | 7 | ≤1 | 5 | 6 | 8 | 8 |
|  | Q88CP1 | Cadmium translocating P-type ATPase | 28 | 25 | 17 | 21 | 22 | 21 | 16 | 13 |
|  | Q88HA5 | Multidrug efflux RND membrane fusion protein | 12 | 4 | 11 | 7 | 7 | 5 | 12 | 6 |
|  | Q88GY1 | Multidrug efflux transport system-putative membrane fusion protein | ≤1 | ≤1 | 4 | ≤1 | 2 | ≤1 | 4 | ≤1 |
|  | P0C070 | Multidrug/solvent efflux pump membrane transporter MepB | 9 | 6 | 7 | 10 | 7 | 5 | 11 | 11 |
|  | P0C071 | Multidrug/solvent efflux pump outer membrane protein MepC | 11 | 14 | 16 | 14 | 16 | 17 | 20 | 15 |
|  | P0C069 | Multidrug/solvent efflux pump periplasmic linker protein MepA | 9 | 5 | 8 | 7 | 6 | 7 | 8 | 8 |
|  | O52248 | Toluene efflux pump membrane transporter TtgB | 9 | 6 | 7 | 10 | 7 | 5 | 11 | 11 |
|  | Q9WWZ8 | Toluene efflux pump outer membrane protein TtgC | 11 | 14 | 16 | 14 | 16 | 17 | 20 | 15 |
|  | Q9WWZ9 | Toluene efflux pump periplasmic linker protein TtgA | 10 | 5 | 8 | 7 | 6 | 7 | 8 | 9 |
|  | Q88Q18 | Endopeptidase La | 16 | 12 | 15 | 9 | 7 | 9 | 12 | 11 |
|  | Q88N31 | Probable efflux pump membrane transporter TtgB | 9 | 6 | 7 | 10 | 7 | 5 | 11 | 11 |
|  | Q88N32 | Probable efflux pump outer membrane protein TtgC | 11 | 14 | 16 | 14 | 16 | 17 | 20 | 15 |
|  | Q88N30 | Probable efflux pump periplasmic linker TtgA | 9 | 5 | 8 | 7 | 6 | 7 | 8 | 8 |
|  |  |  |  |  |  |  |  |  |  |  |
| **Siderophores** | Q88F81 | Outer membrane ferripyoverdine receptor FpvA, TonB-dependent | 5 | 3 | 11 | 10 | ≤1 | ≤1 | 8 | 6 |
|  | Q88HM4 | Putative Outer membrane ferric siderophore receptor | 7 | 7 | 5 | 7 | 8 | 5 | 6 | 5 |
|  | Q88F82 | Pyoverdine ABC export system, fused ATPase and permease components | ≤1 | ≤1 | 2 | 3 | ≤1 | ≤1 | 3 | 2 |
| **DNA repair** | Q88M08 | DNA gyrase subunit A | 23 | 18 | 20 | 19 | 17 | 16 | 19 | 16 |
|  | Q88RW6 | DNA gyrase subunit B | 14 | 18 | 17 | 21 | 14 | 19 | 19 | 18 |
|  | Q88C31 | DNA helicase | 4 | 3 | 4 | 2 | 5 | 2 | 3 | 3 |
|  | Q88GN0 | DNA helicase-related protein | 18 | 12 | 14 | 17 | 7 | 8 | 19 | 15 |
|  | Q88DD1 | DNA mismatch repair protein MutL | 3 | 2 | 4 | 4 | 2 | ≤1 | 7 | 3 |
|  | Q88ME7 | DNA mismatch repair protein MutS | 5 | 3 | 4 | 7 | 3 | 4 | 7 | 7 |
|  | Q88DU0 | DNA repair protein RecN | 3 | 2 | ≤1 | ≤1 | ≤1 | ≤1 | 3 | 2 |
|  |  |  |  |  |  |  |  |  |  |  |
| **Oxidative stress** | Q88NX2 | Glutaredoxin | 7 | 5 | 6 | 5 | 6 | 4 | 6 | 4 |
|  | Q88GA5 | Glutathione reductase | 4 | 3 | 2 | 3 | 3 | 4 | ≤1 | 2 |
|  | Q88LV6 | Glutathione S-transferase family protein | 2 | 2 | ≤1 | 2 | 2 | ≤1 | ≤1 | ≤1 |
|  | Q88K19 | Glutathione S-transferase family protein | 4 | 3 | 4 | 6 | 4 | 3 | 5 | 5 |
|  | Q88GI1 | Glutathione S-transferase family protein | 2 | 3 | 2 | ≤1 | 2 | 2 | ≤1 | 2 |
|  | Q88RE7 | Glutathione S-transferase | 5 | 4 | ≤1 | 5 | 2 | 3 | 3 | 3 |
|  | Q88D35 | Glutathione synthetase | 4 | 5 | 3 | 6 | 4 | 3 | 5 | ≤1 |
|  | Q88QK9 | Catalase | 10 | 11 | 10 | 10 | 6 | 13 | 14 | 13 |
|  | Q88GQ0 | Catalase-peroxidase | 5 | 5 | 2 | 5 | ≤1 | 3 | 4 | 5 |
|  | Q88NW9 | Putative peroxiredoxin | 23 | ≤1 | 22 | 24 | 24 | 23 | 30 | 22 |
|  | Q88R98 | Peroxidase | 3 | 5 | 2 | ≤1 | 4 | 2 | 4 | 2 |
|  | Q88DU1 | Protein GrpE | 7 | 5 | 5 | 7 | 6 | 5 | 4 | 5 |
|  |  |  |  |  |  |  |  |  |  |  |
| **Phosphate metabolism** | Q88CG5 | Exopolyphosphatase | 8 | 11 | 11 | 12 | 8 | 10 | 10 | 6 |
|  | Q88QF6 | Inorganic pyrophosphatase | 12 | 6 | 6 | 9 | 7 | 7 | 10 | 7 |
|  | Q88NC1 | PhoH family protein | 8 | 9 | 6 | 9 | 8 | 9 | 8 | 7 |
|  | Q88FS0 | Phosphatase NudJ | ≤1 | ≤1 | 2 | ≤1 | ≤1 | 2 | 2 | ≤1 |
|  | Q88PS4 | Phosphate acetyltransferase | 4 | 3 | ≤1 | ≤1 | ≤1 | 3 | 4 | 3 |
|  | Q88JJ0 | Phosphate import ATP-binding protein PstB 1 | ≤1 | ≤1 | 7 | 7 | ≤1 | ≤1 | 9 | 10 |
|  | Q88C57 | Phosphate import ATP-binding protein PstB 2 | ≤1 | ≤1 | 3 | 5 | ≤1 | ≤1 | 6 | 8 |
|  | Q88C56 | Phosphate transport system permease protein PstA | ≤1 | ≤1 | ≤1 | 2 | ≤1 | ≤1 | 5 | 2 |
|  | Q88N43 | Phosphate transporter | 2 | 3 | ≤1 | 3 | ≤1 | 3 | 4 | 4 |
|  | Q88FJ2 | Phosphate transporter | ≤1 | ≤1 | ≤1 | ≤1 | ≤1 | ≤1 | 3 | 2 |
|  | Q88JJ3 | Phosphate-binding protein PstS | 5 | 4 | 17 | 20 | 3 | 7 | 24 | 22 |
|  | Q88C58 | Phosphate-specific transport system accessory protein PhoU | 3 | 3 | 5 | 9 | 2 | 4 | 5 | 6 |
|  | Q88P93 | Phospholipid ABC transporter | ≤1 | ≤1 | ≤1 | 2 | 2 | 3 | 6 | 4 |
|  | Q88PM6 | Phosphonate transport system-binding protein | 3 | 2 | 2 | 7 | ≤1 | 3 | 2 | 2 |
|  | Q88PQ5 | Phosphotransferase system, fructose-specific EI/HPr/EIIA components | 12 | 7 | 11 | 8 | 6 | 9 | 6 | 2 |
|  | Q88CG4 | Polyphosphate kinase | 11 | 8 | 12 | 14 | 9 | 13 | 16 | 12 |
|  | Q88EW3 | Protein phosphatase CheZ | 7 | 4 | 4 | 3 | 2 | 4 | 7 | 3 |
|  | Q88P10 | Putative phosphatase | ≤1 | ≤1 | 6 | 7 | ≤1 | ≤1 | 15 | 9 |
|  | Q88C54 | Putative phosphate ABC transporter, periplasmic phosphate-binding protein | 4 | 4 | 16 | 16 | ≤1 | 3 | 23 | 18 |
|  | Q88C55 | Putative phosphate transport system permease protein | ≤1 | ≤1 | ≤1 | 2 | ≤1 | ≤1 | 5 | 7 |
|  | Q88HI1 | Putative phosphonate dehydrogenase | 3 | 5 | ≤1 | 3 | 3 | 3 | ≤1 | ≤1 |
|  |  |  |  |  |  |  |  |  |  |  |
| **Sulfur metabolism** | Q88MC7 | Putative Thioredoxin | 3 | 2 | 3 | ≤1 | 3 | 3 | 2 | 2 |
|  | Q88EZ5 | Sulfate ABC transporter | 4 | 3 | 2 | 7 | 3 | 5 | 4 | 2 |
|  | Q88NA8 | Sulfate adenylyltransferase subunit 1 | 16 | 11 | 14 | 15 | 9 | 12 | 9 | 11 |
|  | Q88NA9 | Sulfate adenylyltransferase subunit 2 | 6 | 7 | 5 | 7 | 5 | 6 | 6 | 5 |
|  | Q88CX5 | Sulfurtransferase | 2 | 4 | 4 | 4 | 2 | ≤1 | 5 | 5 |
|  | Q88PD5 | Superoxide dismutase [Fe] | 10 | 5 | 8 | 5 | 10 | 5 | 10 | 4 |
|  | Q88GY0 | Thiol peroxidase | 13 | 9 | 7 | 5 | 6 | 3 | 8 | 4 |
|  | Q88QI2 | Thioredoxin | 7 | 4 | 6 | 9 | 8 | 7 | 9 | 6 |
|  | Q88CG6 | Thioredoxin | 10 | 10 | 9 | 15 | 11 | 11 | 15 | 8 |
|  | Q88QT9 | Thiosulfate sulfurtransferase GlpE | ≤1 | 4 | ≤1 | 2 | ≤1 | 4 | ≤1 | 3 |
|  |  |  |  |  |  |  |  |  |  |  |
| **Iron metabolism** | Q88KB2 | Fe/S biogenesis protein NfuA | 5 | 5 | 5 | 5 | 7 | 3 | 4 | 2 |
|  | Q88MD5 | Ferredoxin--NADP(+) reductase | 7 | 6 | 5 | 8 | 7 | 10 | 7 | 10 |
|  | Q88DT9 | Ferric uptake regulation protein | 7 | 3 | 5 | 3 | 5 | 4 | 6 | 3 |
|  | Q88PV4 | Ferrochelatase | 3 | ≤1 | ≤1 | 3 | 2 | 3 | 2 | 6 |
|  | Q88NX1 | Bacterioferritin | 9 | 8 | 8 | 9 | 9 | 7 | 10 | 7 |
|  | Q88PK6 | Iron-binding protein IscA | ≤1 | 2 | ≤1 | 2 | 2 | ≤1 | ≤1 | ≤1 |
|  | Q88CF6 | Iron-sulfur cluster assembly protein CyaY | ≤1 | 3 | ≤1 | 2 | ≤1 | 2 | ≤1 | 2 |
|  | Q88PK7 | Iron-sulfur cluster assembly scaffold protein IscU | 5 | 2 | 3 | 2 | 3 | ≤1 | 2 | ≤1 |
|  | Q88NV6 | Iron-sulfur cluster carrier protein | 8 | 3 | 2 | ≤1 | 4 | ≤1 | 4 | 3 |
|  | Q88F40 | Iron-sulfur cluster-binding protein | 8 | 7 | 7 | 9 | 5 | 7 | 8 | 10 |
|  |  |  |  |  |  |  |  |  |  |  |
|  |  |  |  |  |  |  |  |  |  |  |
| **Membrane** | Q88DN0 | LPS-assembly lipoprotein LptE | 11 | 5 | 9 | 10 | 8 | 5 | 11 | 6 |
|  | A0A140FVZ0 | LPS-assembly protein LptD | 25 | 15 | 24 | 21 | 23 | 17 | 31 | 17 |
|  | Q88KG8 | Major outer membrane lipoprotein | 11 | 7 | 8 | 7 | 7 | 8 | 13 | 9 |
|  | Q88NT3 | OmpA family protein | ≤1 | ≤1 | 2 | 2 | 2 | 3 | 2 | 2 |
|  | Q88MR7 | OmpA family protein | 2 | 2 | 2 | 2 | 2 | 3 | 2 | 2 |
|  | Q88FA0 | OmpA family protein | ≤1 | ≤1 | 2 | ≤1 | ≤1 | 3 | 2 | 3 |
|  | Q88PS5 | OmpA/MotB domain-containing protein | 11 | 9 | 7 | 8 | 9 | 9 | 11 | 10 |
|  | Q88PS5 | OmpA/MotB domain-containing protein | 11 | 9 | 7 | 8 | 9 | 9 | 11 | 10 |
|  | Q88DI7 | Outer membrane copper receptor OprC | 33 | ≤1 | 17 | 23 | 20 | 22 | 17 | 16 |
|  | Q88DA4 | Outer membrane efflux protein | 12 | 9 | 8 | 8 | 13 | 6 | 10 | 7 |
|  |  |  |  |  |  |  |  |  |  |  |
|  | Q88NS3 | Outer membrane lipoprotein | 8 | 7 | 8 | 5 | 5 | 7 | 6 | 8 |
|  | Q88NM2 | Outer membrane protein H1 | 28 | 21 | 40 | ≤1 | 26 | 23 | 52 | 37 |
|  | Q88RL3 | Putative lipoprotein | ≤1 | 4 | 3 | 4 | 4 | 2 | 4 | 5 |
|  | Q88PU5 | Putative Lipoprotein | 9 | 5 | 5 | 6 | 8 | 10 | 10 | 9 |
|  | Q88NQ2 | Putative lipoprotein | ≤1 | 3 | 2 | 2 | 2 | 2 | ≤1 | 2 |
|  | Q88NH1 | Putative Lipoprotein | 8 | 7 | 10 | 8 | 6 | 7 | 10 | 8 |
|  | Q88N90 | Putative Lipoprotein | 7 | 6 | 4 | 4 | 5 | 5 | 6 | 6 |
|  | Q88N87 | Putative lipoprotein | 13 | 10 | 15 | 16 | 9 | 10 | 17 | 12 |
|  | Q88F99 | Putative Lipoprotein | 4 | 3 | 4 | 4 | 3 | 4 | 2 | 2 |
|  | Q88C79 | Putative Lipoprotein | 5 | 4 | 6 | 5 | 5 | 4 | 5 | 6 |
|  | A0A140FWL3 | Putative lipoprotein | 11 | 11 | 8 | 9 | 6 | 14 | 8 | 10 |
|  | H2EPL0 | Putative major outer membrane lipoprotein | 8 | 6 | 5 | 5 | 2 | 3 | 6 | 3 |
|  |  |  |  |  |  |  |  |  |  |  |
| **Other** | Q88MC8 | Arsenate reductase | 3 | 6 | 3 | 6 | 4 | 7 | 4 | 5 |
|  | Q88DF5 | Azurin | 4 | 5 | 7 | 5 | 5 | 4 | 5 | 6 |
|  | Q88NV5 | Cold shock protein CapB | 18 | 16 | 17 | 24 | 15 | 21 | 13 | 21 |
|  | Q88HW9 | Heat shock protein, HSP20 family | 10 | 11 | 8 | 9 | 10 | 6 | 9 | 7 |
|  | Q8KQ23 | PHA synthase 1 | ≤1 | 6 | 6 | 8 | 6 | 5 | ≤1 | 8 |
|  | Q88KV2 | Universal stress protein family | 10 | 8 | 8 | 12 | 8 | 10 | 16 | 9 |
|  | Q88JK1 | Universal stress protein family | 18 | 17 | 15 | 21 | 15 | 19 | 19 | 18 |
|  | Q88JA4 | Universal stress protein family | 3 | 4 | 2 | 4 | 3 | 2 | 3 | 2 |
|  | Q88HR5 | Universal stress protein family | 7 | 6 | 6 | 9 | 7 | 5 | 5 | 3 |
|  | Q88L05 | Universal stress protein | 4 | 4 | 3 | 4 | 3 | 3 | 3 | 2 |
|  |  |  |  |  |  |  |  |  |  |  |
| **Plasmid encoded efflux pump** | Q58AJ6 | P-type ATPase involved in Pb(II) resistance PbrA | ≤1 | ≤1 | ≤1 | ≤1 | ≤1 | ≤1 | 4 | 3 |

| ***Variovorax paradoxus* B4** |  |  | Pb (mM) | | | |
| --- | --- | --- | --- | --- | --- | --- |
|  | Code | function | **0** | **0** | **1** | **1** |
| **Chaperone** | T1XCK5 | 33 kDa chaperonin | 2 | 4 | 3 | 3 |
|  | T1X7Z8 | 60 kDa chaperonin | 78 | 95 | 70 | 94 |
|  | T1XKS6 | 60 kDa chaperonin | 78 | 95 | 9 | 94 |
|  | Q88Q71 | Chaperone protein ClpB | 8 | 8 | 3 | ≤1 |
|  | T1XA40 | Chaperone protein ClpB | 27 | 31 | 28 | 33 |
|  | T1X8N1 | Chaperone protein DnaJ | 4 | 4 | 2 | ≤1 |
|  | T1X996 | Chaperone protein DnaK | 20 | 28 | 25 | 21 |
|  | T1XAR1 | Chaperone protein HscA homolog | 4 | ≤1 | 6 | 4 |
|  | T1XJD1 | Chaperone protein HtpG | 26 | 20 | 22 | 21 |
|  | T1XI04 | Chaperone SurA | 11 | 12 | 13 | 14 |
|  | T1XA42 | Co-chaperone protein HscB homolog | ≤1 | ≤1 | 2 | ≤1 |
|  |  |  |  |  |  |  |
| **Efflux** | T1XHR1 | Efflux pump membrane transporter | 8 | 8 | 5 | 3 |
|  | T1XC33 | Efflux pump membrane transporter | ≤1 | ≤1 | 2 | ≤1 |
|  | T1XC76 | Efflux transporter, RND family | 6 | 4 | 3 | 3 |
|  | T1XL89 | Efflux transporter, RND family | 6 | 5 | 4 | 5 |
|  | T1XB49 | Putative multidrug efflux transporter, AcrB/AcrD/AcrF family | 6 | 8 | 2 | 3 |
|  | T1XDF6 | Putative multidrug efflux transporter, AcrB/AcrD/AcrF family | 2 | 3 | 3 | 4 |
|  | T1XDX1 | Cobalt-zinc-cadmium resistance protein CzcA | 4 | 6 | 2 | 4 |
|  | T1X8G1 | Putative TRAP dicarboxylate transporter, subunit DctP | 26 | 29 | 21 | 24 |
|  | T1X8D4 | Putative TRAP dicarboxylate transporter, subunit DctP | 6 | 6 | 6 | 7 |
|  | T1XI44 | Putative TRAP dicarboxylate transporter, subunit DctP | 11 | 12 | 14 | 13 |
|  | T1XMC3 | Putative TRAP dicarboxylate transporter, subunit DctP | 2 | ≤1 | 2 | ≤1 |
|  | T1XKY1 | Putative TRAP transporter, DctP subunit | 4 | 7 | 5 | 8 |
|  | T1XED5 | TRAP dicarboxylate transporter, subunit DctP | 11 | 9 | 9 | 10 |
|  | T1XDV3 | TRAP dicarboxylate transporter, subunit DctP | 5 | 6 | 7 | 7 |
|  | T1X4X2 | TRAP dicarboxylate transporter, subunit DctP | 6 | 5 | 3 | 5 |
|  | T1X610 | TRAP dicarboxylate transporter, subunit DctP | 15 | 15 | 8 | 8 |
|  | T1XBY7 | Putative heavy metal translocating P-type ATPase | ≤1 | ≤1 | ≤1 | 2 |
|  | T1XK38 | Putative efflux transporter, RND family | 3 | 2 | ≤1 | 2 |
|  |  |  |  |  |  |  |
| **Siderophores** | T1XEH6 | TonB-dependent siderophor receptor | ≤1 | ≤1 | 20 | 18 |
|  | T1X5P8 | TonB-dependent siderophore receptor | ≤1 | ≤1 | 17 | 18 |
|  | T1X6U1 | TonB-dependent siderophore receptor | ≤1 | ≤1 | 22 | 21 |
|  | T1XF69 | TonB-dependent siderophore receptor | ≤1 | ≤1 | 16 | 23 |
|  | T1XI89 | TonB-dependent siderophore receptor | ≤1 | ≤1 | 8 | 5 |
|  | T1XHM7 | TonB-dependent siderophore receptor | 4 | ≤1 | 8 | 9 |
|  | T1XC46 | Putative TonB-dependent receptor | 35 | 31 | 23 | 25 |
|  |  |  |  |  |  |  |
| **DNA repair** | T1XEN2 | DNA mismatch repair protein MutS | 5 | 3 | ≤1 | 2 |
|  |  |  |  |  |  |  |
| **Oxidative stress** | T1X484 | Catalase | 5 | 3 | 5 | 5 |
|  | T1XD68 | Catalase-peroxidase | 18 | 21 | 17 | 21 |
|  | T1XFZ6 | Glutaredoxin | 3 | 3 | 2 | 5 |
|  | T1X421 | Glutathione synthetase | 5 | 5 | 4 | 4 |
|  | T1XBH8 | Glutathione-binding protein | 16 | 12 | 18 | 20 |
|  | T1XI47 | Glutathione-disulfide reductase Gor | 4 | 2 | 2 | 3 |
|  | T1XIJ8 | Peroxiredoxin | 9 | 7 | 6 | 7 |
|  | T1XED2 | Superoxide dismutase [Cu-Zn] | 6 | 5 | 13 | 5 |
|  | T1XAP3 | Superoxide dismutase | 17 | 16 | 9 | 13 |
|  | T1XI85 | Superoxide dismutase | 14 | 17 | 10 | ≤1 |
|  | T1XIV7 | Glutathione transport system, substrate binding protein GsiB | 3 | ≤1 | ≤1 | 3 |
|  | T1XEI0 | Glutaredoxin | 3 | ≤1 | ≤1 | 2 |
|  |  |  |  |  |  |  |
| **Phosphate metabolism** | T1X8Z6 | PhoH-like protein | 16 | 13 | 12 | 13 |
|  | T1XH38 | PhoH-like protein | 6 | 2 | 2 | 3 |
|  | T1XBL7 | Phosphate-specific transport system accessory protein PhoU | 3 | 2 | 2 | 4 |
|  | T1XA01 | Putative phosphonate ABC transporter, phosphonte-binding protein | 6 | 5 | 3 | 7 |
|  | T1XCW6 | Putative sulfatase | ≤1 | ≤1 | 2 | ≤1 |
|  | T1XBK9 | Putative sulfate ABC transporter, sulfate-binding protein CysP | 3 | 7 | 4 | 8 |
|  | T1XCU4 | Uracil phosphoribosyltransferase | 9 | 7 | 8 | 7 |
|  |  |  |  |  |  |  |
| **Iron metabolism** | T1X407 | Ferredoxin-NADP reductase Fpr | 8 | 14 | 10 | 11 |
|  | T1X5M1 | Ferric uptake regulation protein | 4 | 5 | 4 | 5 |
|  | T1XIB9 | Ferritin-like domain-containing protein | 5 | 4 | 5 | 3 |
|  | T1X7Y1 | Ferrous iron transport protein B | 2 | ≤1 | 8 | 8 |
|  | T1X6W0 | Iron-sulfur cluster assembly protein CyaY | ≤1 | 4 | 3 | 2 |
|  | T1XAA1 | Iron-sulfur cluster assembly scaffold protein IscU | ≤1 | ≤1 | 3 | 3 |
|  | T1XFF8 | Iron-sulfur cluster carrier protein | 11 | 9 | 7 | 4 |
|  | T1XBJ2 | Probable Fe (2+)-trafficking protein | 5 | 2 | 2 | 2 |
|  | T1XEG8 | Putative ABC transporter, iron-binding protein | 6 | 7 | 8 | 12 |
|  | T1XGT3 | Putative bacterioferritin | 6 | 7 | 2 | 3 |
|  | T1XBU5 | Putative iron-containing alcohol dehydrogenase | 3 | 5 | 4 | 4 |
|  | T1XH48 | Putative iron-sulfur cluster insertion protein ErpA | ≤1 | 2 | 2 | 2 |
|  |  |  |  |  |  |  |
| **Sulfur metbolism** | T1XBW9 | Sulfate adenylyltransferase, subunit 1 CysN | 2 | ≤1 | 3 | 3 |
|  | T1X517 | Thiol:disulfide interchange protein | 7 | 7 | 5 | 8 |
|  | T1XHM6 | Thiol:disulfide interchange protein | 5 | 5 | 6 | 8 |
|  | T1XJN2 | Thiolase | ≤1 | 2 | 2 | 5 |
|  | T1X9Y4 | Thioredoxin domain-containing protein | ≤1 | 2 | 2 | ≤1 |
|  | T1XAJ2 | Thioredoxin | 7 | 8 | 5 | 7 |
|  | T1XHF9 | Thioredoxin | 7 | 8 | 7 | 6 |
|  | T1X8H6 | Thioredoxin reductase | 8 | 8 | 7 | 10 |
|  | T1XBZ2 | Sulfate ABC transporter, sulfate-binding protein | ≤1 | 3 | ≤1 | 3 |
|  |  |  |  |  |  |  |
| **Membrane** | T1X4X7 | LPS-assembly lipoprotein LptE | 4 | 3 | 6 | 4 |
|  | T1XIZ2 | LPS-assembly protein LptD | 11 | 9 | 10 | 9 |
|  | T1X777 | Omega-amino acid--pyruvate aminotransferase | 2 | ≤1 | 2 | 2 |
|  | T1XIM6 | OmpA family protein | 8 | 6 | 7 | 5 |
|  | T1XHL2 | OmpA family protein | 4 | 2 | 4 | 2 |
|  | T1XJM4 | OmpA family protein | 3 | ≤1 | 5 | 2 |
|  | T1XEI3 | OmpA/MotB domain-containing protein | 17 | 13 | 13 | 13 |
|  | T1X8D2 | Outer membrane protein, OmpW family | 5 | 6 | 6 | 7 |
|  | T1X5I2 | Outer memprane protein, OmpW family | ≤1 | ≤1 | 2 | 3 |
|  |  |  |  |  |  |  |
| **Other** | T1XDX0 | Putative multidrug resistance protein A | 2 | ≤1 | 3 | 3 |
|  | T1XKE8 | Putative multidrug resistance protein | 5 | 2 | 4 | 5 |
|  | T1XJX7 | Putative universal stress protein | 7 | 6 | 6 | 6 |
|  | T1XL74 | Putative universal stress protein | 4 | 5 | 5 | 5 |
|  | T1XL79 | Putative universal stress protein | 12 | 11 | 13 | 8 |
|  | T1XJ74 | Putative universal stress protein | 10 | 11 | 10 | 8 |
|  | T1XKY3 | Putative universal stress protein | 7 | 7 | 7 | 9 |
|  | T1XK86 | Putative universal stress protein | 3 | 7 | 3 | 6 |
|  | T1XLM7 | Putative universal stress protein | 6 | 6 | 9 | 4 |
|  | T1XLN3 | Putative universal stress protein | 10 | 9 | 9 | 11 |
|  | T1XBW3 | Putative heat shock protein DnaJ | ≤1 | 2 | ≤1 | 2 |

| ***Delftia acidovorans* SPH-1** |  |  | Pb (mM) | | | |
| --- | --- | --- | --- | --- | --- | --- |
|  | **Code** | **Function** | **0** | **0** | **1** | **1** |
| **Chaperone** | A9BXL2 | 10 kDa chaperonin | 7 | 8 | 13 | 8 |
|  | A9BXL3 | 60 kDa chaperonin | 63 | 69 | 66 | 76 |
|  | A9BNE1 | Chaperone protein ClpB | 18 | 14 | 16 | 28 |
|  | A9BNG6 | Chaperone protein DnaJ | 8 | 7 | 8 | 8 |
|  | A9BNG5 | Chaperone protein DnaK | 23 | 19 | 28 | 29 |
|  | A9BWU9 | Chaperone protein HscA homolog | 4 | 4 | 10 | 10 |
|  | A9BUB7 | Chaperone protein HtpG | 20 | 16 | 27 | 18 |
|  | A9C1J9 | Chaperone SurA | 7 | 7 | 12 | 10 |
|  | A9BMM3 | Outer membrane chaperone Skp (OmpH) | 6 | 7 | 8 | 9 |
|  | A9BS91 | Molecular chaperone, HSP70 class | ≤1 | ≤1 | ≤1 | 3 |
|  |  |  |  |  |  |  |
| **Efflux** | A9BR88 | Efflux pump membrane transporter | 29 | 25 | 30 | 23 |
|  | A9BXU3 | Efflux pump membrane transporter | ≤1 | ≤1 | 2 | ≤1 |
|  | A9BPA2 | Efflux transporter, RND family, MFP subunit | 7 | 5 | 12 | 13 |
|  | A9BR62 | Efflux transporter, RND family, MFP subunit | ≤1 | ≤1 | ≤1 | 3 |
|  | A9BR89 | Efflux transporter, RND family, MFP subunit | 23 | 21 | 33 | 30 |
|  | A9BSB9 | Efflux transporter, RND family, MFP subunit | 5 | 3 | 3 | 2 |
|  | A9BUT5 | Efflux transporter, RND family, MFP subunit | ≤1 | ≤1 | 3 | 2 |
|  | A9BTI7 | Efflux transporter, RND family, MFP subunit | ≤1 | ≤1 | 3 | 2 |
|  | A9BZU7 | Efflux transporter, RND family, MFP subunit | 2 | ≤1 | 3 | ≤1 |
|  | A9BPA1 | Heavy metal efflux pump, CzcA family | 4 | 3 | 13 | 10 |
|  | A9BR61 | Heavy metal efflux pump, CzcA family | ≤1 | ≤1 | 3 | 2 |
|  | A9BSB8 | Heavy metal efflux pump, CzcA family | ≤1 | ≤1 | 2 | ≤1 |
|  | A9BUT6 | Heavy metal efflux pump, CzcA family | ≤1 | ≤1 | 5 | 2 |
|  | A9BR67 | Heavy metal translocating P-type ATPase | ≤1 | ≤1 | 25 | 19 |
|  | A9BZF3 | Heavy metal translocating P-type ATPase | ≤1 | ≤1 | 5 | 3 |
|  | A9BPB8 | Heavy metal transport/detoxification protein | 3 | 4 | 3 | 3 |
|  | A9BQR9 | RND efflux system, outer membrane lipoprotein, NodT family | 6 | 7 | 10 | 9 |
|  | A9BR87 | RND efflux system, outer membrane lipoprotein, NodT family | 19 | 19 | 23 | 19 |
|  | A9BRZ7 | RND efflux system, outer membrane lipoprotein, NodT family | 2 | 4 | 3 | 2 |
|  | A9BTI5 | RND efflux system, outer membrane lipoprotein, NodT family | 3 | ≤1 | ≤1 | 2 |
|  | A9BYZ0 | TRAP dicarboxylate transporter, DctM subunit | 2 | 2 | 2 | ≤1 |
|  | A9BSJ9 | TRAP dicarboxylate transporter, DctP subunit | 11 | 12 | 8 | 10 |
|  | A9C2P7 | TRAP dicarboxylate transporter, DctP subunit | 3 | 3 | 4 | 3 |
|  | A9C1A8 | TRAP dicarboxylate transporter, DctP subunit | 3 | ≤1 | 2 | ≤1 |
|  | A9BYY8 | TRAP dicarboxylate transporter-DctP subunit | 16 | 17 | 13 | 16 |
|  | A9BYY9 | TRAP dicarboxylate transporter-DctP subunit | 19 | 18 | 13 | 13 |
|  |  |  |  |  |  |  |
| **Siderophores** | A9C3E0 | TonB family protein | ≤1 | ≤1 | 2 | 2 |
|  | A9BQT2 | TonB-dependent hemoglobin/transferrin/lactoferrin family receptor | ≤1 | ≤1 | 9 | 4 |
|  | A9C2L3 | TonB-dependent receptor | 2 | 2 | ≤1 | 3 |
|  | A9C2M1 | TonB-dependent receptor | ≤1 | 3 | 7 | 9 |
|  | A9BUB5 | TonB-dependent siderophore receptor | 6 | 5 | 12 | 13 |
|  | A9BZ11 | TonB-dependent siderophore receptor | 13 | 7 | 19 | 10 |
|  | A9BVM4 | TonB-dependent siderophore receptor | ≤1 | ≤1 | ≤1 | 4 |
|  | A9BZJ1 | TonB-dependent siderophore receptor | 3 | ≤1 | 2 | 4 |
|  | A9C266 | TonB-dependent siderophore receptor | ≤1 | ≤1 | 5 | 4 |
|  | A9BM36 | TonB-dependent siderophore receptor | ≤1 | ≤1 | 10 | 6 |
|  | A9BM70 | TonB-dependent siderophore receptor | ≤1 | ≤1 | 3 | 2 |
|  | A9C157 | TonB-dependent siderophore receptor | ≤1 | ≤1 | ≤1 | 2 |
|  |  |  |  |  |  |  |
|  |  |  |  |  |  |  |
| **DNA repair** | A9BN65 | DNA mismatch repair protein MutL | ≤1 | 2 | 2 | ≤1 |
|  | A9BTE6 | DNA mismatch repair protein MutS | ≤1 | ≤1 | 2 | ≤1 |
|  |  |  |  |  |  |  |
| **Oxidative stress** | A9BLW0 | Catalase | 13 | 9 | 16 | 6 |
|  | A9BP62 | Glutaredoxin | 2 | 2 | 2 | 2 |
|  | A9BQI9 | Glutathione S-transferase domain | 3 | 2 | 3 | 4 |
|  | A9BQ87 | Glutathione S-transferase domain | 4 | 3 | 6 | ≤1 |
|  | A9BYB1 | Glutathione S-transferase domain | 4 | 4 | 2 | 2 |
|  | A9BWS5 | Glutathione S-transferase domain | ≤1 | ≤1 | 3 | 2 |
|  | A9BMC1 | Glutathione S-transferase domain | 4 | 4 | 5 | 4 |
|  | A9BX53 | Glutathione S-transferase domain | 7 | 8 | 13 | 11 |
|  | A9BXQ4 | Glutathione S-transferase domain | 4 | 4 | 2 | ≤1 |
|  | A9C1F9 | Glutathione synthetase | 3 | ≤1 | 2 | ≤1 |
|  | A9BTM6 | Glutathione-disulfide reductase | 3 | 4 | 6 | 6 |
|  | A9BQH5 | Glyceraldehyde-3-phosphate dehydrogenase | 11 | 10 | 13 | 8 |
|  | A9BNG4 | Protein GrpE | 2 | 2 | 5 | 2 |
|  | A9BWP6 | Superoxide dismutase [Cu-Zn] | 4 | 7 | 3 | 3 |
|  | A9C1X2 | Superoxide dismutase | ≤1 | ≤1 | 5 | 4 |
|  | A9BWI8 | Superoxide dismutase | 11 | 17 | 13 | 14 |
|  |  |  |  |  |  |  |
| **Phosphate metabolism** | A9BXS8 | Carbamoyl-phosphate synthase L chain ATP-binding | 10 | 11 | 17 | 18 |
|  | A9C1D5 | Carbamoyl-phosphate synthase L chain ATP-binding | 17 | 15 | 20 | 17 |
|  | A9BML5 | Carbamoyl-phosphate synthase large chain | 23 | 25 | 24 | 29 |
|  | A9BML6 | Carbamoyl-phosphate synthase small chain | 6 | 9 | 9 | 7 |
|  | A9BUG6 | PhoH family protein | 7 | 4 | 8 | 3 |
|  | A9BNF7 | PhoH family protein | 11 | 18 | 16 | 14 |
|  | A9C1H8 | Phosphatase NudJ | 2 | 2 | 3 | ≤1 |
|  | A9BRZ1 | Phosphate acetyltransferase | 9 | 8 | 8 | 11 |
|  | A9BNK7 | Phosphate acyltransferase | 2 | 4 | 5 | 5 |
|  | A9BMJ9 | Phosphate import ATP-binding protein PstB | ≤1 | ≤1 | 6 | 6 |
|  | A9BMK2 | Phosphate-binding protein PstS | 8 | 7 | 19 | 20 |
|  | A9BMJ8 | Phosphate-specific transport system accessory protein PhoU | ≤1 | 2 | 7 | 8 |
|  | A9BZR8 | Phosphonate ABC transporter, periplasmic phosphonate-binding protein | 6 | 8 | 5 | 7 |
|  |  |  |  |  |  |  |
| **Sulfur metabolism** | A9C3A8 | Sulfate ABC transporter, periplasmic sulfate-binding protein | 4 | 6 | 5 | 8 |
|  | A9C3H3 | Sulfate adenylyltransferase | 3 | 3 | 5 | 4 |
|  | A9C3H4 | Sulfate adenylyltransferase, large subunit | 3 | 3 | 8 | 3 |
|  | A9C3A5 | Sulfate/thiosulfate import ATP-binding protein CysA | 5 | 2 | 4 | 7 |
|  | A9BYY2 | Sulfurtransferase | 2 | 3 | 4 | 4 |
|  | A9BM43 | Thioesterase | ≤1 | ≤1 | 5 | 2 |
|  | A9BPI6 | Thioesterase superfamily protein | ≤1 | 2 | 2 | 2 |
|  | A9BRF6 | Thiol:disulfide interchange protein | 7 | 7 | 7 | 4 |
|  | A9BUR7 | Thiol:disulfide interchange protein | 5 | 4 | 5 | 9 |
|  | A9BRS2 | Thioredoxin | 6 | 6 | 5 | 5 |
|  | A9BMI9 | Thioredoxin | 5 | 5 | 6 | 6 |
|  | A9BNU8 | Thioredoxin reductase | 5 | 3 | 6 | 6 |
|  |  |  |  |  |  |  |
| **Iron metabolism** | A9BWN5 | (2Fe-2S)-binding domain protein | 2 | ≤1 | 3 | 4 |
|  | A9BTY1 | Bacterioferritin | ≤1 | 2 | 6 | 5 |
|  | A9BN07 | Bacterioferritin | 2 | 2 | 3 | 3 |
|  | A9BRP4 | Ferredoxin | 2 | 2 | 2 | ≤1 |
|  | A9BTZ6 | Ferredoxin-dependent glutamate synthase | 2 | 4 | 3 | 4 |
|  | A9C1H4 | Ferric uptake regulation protein | 5 | 5 | 5 | 6 |
|  | A9BRU3 | Ferritin Dps family protein | 8 | 8 | 8 | 11 |
|  | A9BR70 | Iron permease FTR1 | ≤1 | ≤1 | 6 | 7 |
|  | A9BXD6 | Iron-sulfur cluster assembly protein CyaY | 3 | ≤1 | 4 | 5 |
|  | A9C062 | Iron-sulfur cluster assembly protein IscA | ≤1 | ≤1 | 3 | 2 |
|  | A9C063 | Iron-sulfur cluster assembly scaffold protein IscU | 3 | 4 | 4 | 3 |
|  | A9BV24 | Iron-sulfur cluster carrier protein | 3 | 2 | 5 | 3 |
|  | A9C177 | Putative iron-sulfur cluster insertion protein ErpA | ≤1 | 2 | 2 | 2 |
|  |  |  |  |  |  |  |
| **Membrane** | A9BVB4 | Basic membrane lipoprotein | 11 | 14 | 11 | 11 |
|  | A9BVB8 | Basic membrane lipoprotein | 19 | 19 | 20 | 20 |
|  | A9BVC2 | Basic membrane lipoprotein | ≤1 | 2 | 2 | 2 |
|  | A9C1G7 | LPS-assembly lipoprotein LptE | 3 | 3 | 4 | 3 |
|  | A9BS80 | OmpA/MotB domain protein | 3 | 3 | 4 | 2 |
|  | A9BWP4 | OmpA/MotB domain protein | 6 | 7 | 8 | 8 |
|  | A9BM02 | OmpA/MotB domain protein | 13 | 14 | 24 | 17 |
|  | A9BRQ8 | OmpW family protein | 4 | 4 | 2 | 3 |
|  | A9BMC0 | OmpW family protein | 13 | 9 | 6 | 14 |
|  | A9BPA0 | Outer membrane efflux protein | 5 | 6 | 10 | 11 |
|  | A9BMM4 | Outer membrane protein assembly factor BamA | 20 | 24 | 27 | 33 |
|  | A9BMV0 | Outer membrane protein assembly factor BamB | 12 | 7 | 11 | 8 |
|  | A9BWB6 | Outer membrane protein assembly factor BamD | 6 | 5 | 3 | 4 |
|  | A9C1H3 | Outer membrane protein assembly factor BamE | 5 | 7 | 5 | 5 |
|  | A9BNU6 | Outer-membrane lipoprotein carrier protein | 2 | 2 | 4 | ≤1 |
|  |  |  |  |  |  |  |
| **Other** | A9BLR8 | Azurin | 7 | 5 | 6 | 5 |
|  | A9BPX2 | Copper resistance protein CopC | 5 | 4 | 4 | 4 |
|  | A9BVF2 | Heat shock protein HSP20 | ≤1 | ≤1 | ≤1 | 3 |
|  | Q88JK1 | Universal stress protein family | ≤1 | ≤1 | 2 | ≤1 |
|  | A9BVP7 | Universal stress protein | 9 | 8 | 13 | 8 |
|  | A9BX02 | Universal stress protein | 7 | 10 | 8 | 7 |

| **Co-culture** | Proteins identified constituting libraries | Quantified proteins in SWATH samples | SWATH Proteome coverage |
| --- | --- | --- | --- |
|  |  |  |  |
| *Pseudomonas putida* KT2440 | 3123 | 1693 | 28.5 |
| *Delftia acidovorans* SPH-1 | 3397 | 1895 | 31.7 |
|  |  |  |  |
| *Pseudomonas putida* KT2440 | 3206 | 1708 | 28.7 |
| *Variovorax paradoxus* B4 | 4046 | 2037 | 30.1 |

**Table S7:** Overview of percentage of proteome coverage

**Table S8:** *p*-values associated with conjugation involved proteins as represented in Figure 6 (cell normalized). *p*-values were calculated with protein log-2 abundances using a t-test. *p-*value < 0,05 were considered (grey dashed cells).
